# Effect of the data-informed platform for health intervention on the culture of data use for decision-making among district health office staff in North Shewa Zone, Ethiopia: a cluster-randomised controlled trial

**DOI:** 10.1186/s12911-024-02597-x

**Published:** 2024-07-05

**Authors:** Girum Taye Zeleke, Bilal Iqbal Avan, Mehret Amsalu Dubale, Joanna Schellenberg

**Affiliations:** 1 Health System and reproductive health research directorate, Ethiopian Public Health Institute, Addis Ababa, Ethiopia; 2 London School of Hygiene and Tropical Medicine, London, UK

**Keywords:** Data-Informed, Decision-making, District, Ethiopia, Health-office, Staff-culture

## Abstract

**Background:**

Similar to other low and middle-income countries, Ethiopia faces limitations in using local health data for decision-making.We aimed to assess the effect of an intervention, namely the data-informed platform for health, on the culture of data-based decision making as perceived by district health office staff in Ethiopia’s North Shewa Zone.

**Methods:**

By designating district health offices as ‘clusters’, a cluster-randomised controlled trial was implemented. Out of a total of 24 districts in the zone, 12 districts were allocated to intervention arm and the other 12 in the control group arms. In the intervention arm district health office teams were supported in four-monthly cycles of data-driven decision-making over 20 months. This support included: (a) defining problems using a health system framework; (b) reviewing data; (c) considering possible solutions; (d) value-based prioritizing; and (e) a consultative process to develop, commit to, and follow up on action plans. To measure the culture of data use for decision-making in both intervention and control arms, we interviewed 120 health management staff (5 per district office). Using a Likert scale based standard Performance of Routine Information System Management tool, the information is categorized into six domains:- evidence-based decision making, emphasis on data quality, use of information, problem solving, responsibility and motivation. After converting the Likert scale responses into percentiles, difference-in-difference methods were applied to estimate the net effect of the intervention. In intervention districts, analysis of variance was used to summarize variation by staff designation.

**Results:**

The overall decision-making culture in health management staff showed a net improvement of 13% points (95% C.I:9, 18) in intervention districts. The net effect of each of the six domains in turn was an 11% point increase (95% C.I:7, 15) on culture of evidence based decision making, a 16% point increase (95% C.I:8, 24) on emphasis on data quality, a 20% point increase (95% C.I:12, 28) on use of information, a 21% point increase (95% C.I:13, 29) on problem solving, and a 10% point increase (95% C.I:4, 16) on responsibility and motivation. In terms of variation by staff designation within intervention districts, statistically significant differences were observed only for problem solving and responsibility.

**Conclusion:**

The data-informed platform for health strategy resulted in a measurable improvement in data use and structured decision-making culture by using existing systems, namely the Performance Monitoring Team meetings. The intervention supported district health offices in identifying and solving problems through a structured process. After further research, DIPH intervention could also be applied to other health administration and facility levels.

**Trial registration:**

ClinicalTrials.gov ID: NCT05310682, Dated 25/03/ 2022.

**Supplementary Information:**

The online version contains supplementary material available at 10.1186/s12911-024-02597-x.

## Background

In low and middle-income countries like Ethiopia, valuable data from Health Management Information System (HMIS) and other local sources remains underutilized for critical tasks like decision making, health system performance assessment, and district-level planning. [1–3]. Much is known regarding health system data collection and how to improve data quality [4] but less has been documented on how the available data are used to inform decisions. Although data quality is improving [5], health-related programs frequently fall short of efficient use of data for decision making. Data are primarily available in databases and reports and are not sufficiently used to inform program development and improvement, policy development, strategic planning, or advocacy [6].

Although the HMIS is the backbone of a strong health system, studies in Sub-Saharan Africa have indicated gaps in data quality, including completeness & timeliness, accuracy, consistency and optimal utilization of HMIS tools. These could compromise the quality of routine information and limit data utilization for decision-making in the health sector. A study in Illu Aba Bora Zone, Ethiopia also revealed that only half of study participants had good knowledge and a favorable attitude towards the use of existing District Health Information System (DHIS-2) data for decision making [7, 8].

Evidence-based public health decision making is critical for the likelihood of successful health programs & policies, greater workforce productivity, access to higher quality information and more efficient use of public and private resources [9].

Despite global recognition of the need for data-informed decision making at local levels to improve health [10], little is known on how decision making and data use could be interlinked and attained at the district level health system of Ethiopia. Recent technological advancements in computerization of health information functions, such as DHIS2, in Ethiopia and elsewhere, have brought about some progress in data quality improvement and flow at different levels of the health system [11]. In line with this, one of the key priorities of the Ethiopian Ministry of Health (MoH) transformation agenda is changing the culture of information use throughout the continuum of the national health system. The Performance Monitoring Team (PMT) meeting, which happens at district health offices on a monthly basis comprising of multidisciplinary health staff, is a platform for improving use of locally collected data for evidence-based decision making [12]. Despite monthly PMTs, recent analysis conducted in Amhara region North West Ethiopia reported low use of routine health information for decision making [13].

To fill this gap, the Data-Informed Platform for Health (DIPH) approach was implemented in North Shewa Zone, Amhara Region of Ethiopia, to enhance interaction between district-level administration health staff with the aim of coordinating decision-making and planning, and strengthening health systems through capacity-building and effective use of data.

The primary results of the trial are reported elsewhere [14]. In this paper, we report a quantitative study that assessed the effect of DIPH strategy on the culture of data use for decision-making among district health office staff in North Shewa Zone.

## Methods

### Study design

A cluster-randomised controlled trial with pre and post-comparison design was applied. Study districts were considered as ‘clusters’ and equal number of district health offices were allocated to intervention and control arms. The study adheres to CONSORT guidelines and the 2010 checklist of information for a cluster randomised controlled trial is attached as an additional file (CONSORT_Checklist (File 2)).

We used an action research approach to adapt, implement and evaluate the DIPH strategy in a series of phases, and the protocol is attached as an additional file (Protocol (File 1) for details).

**Fig. 1:**
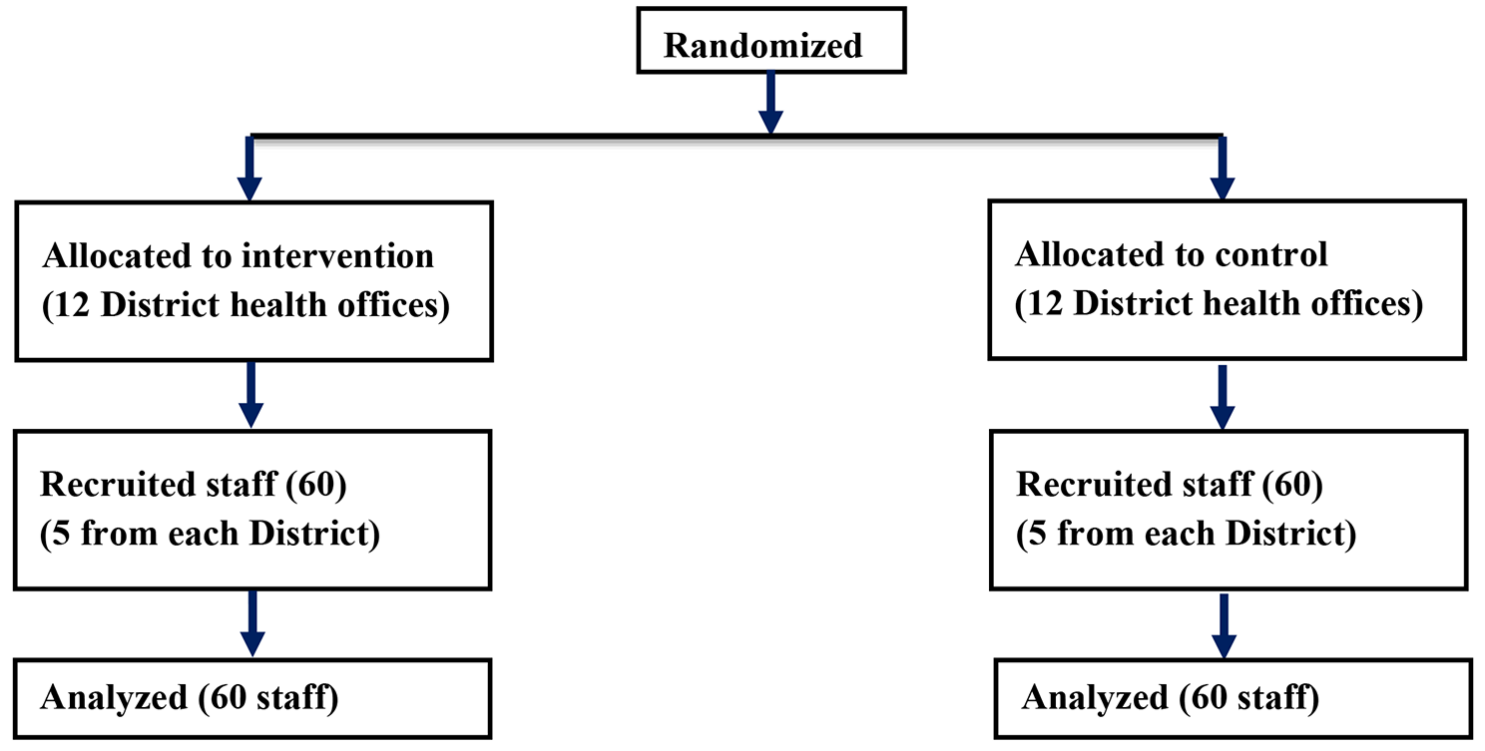
Flow chart of district health office and staff enrolled

Prior to designing DIPH intervention a formative qualitative assessment was done. This assessment found that, although there were diverse data sources including health information system data, disease & emergency surveillance data and additional disease or program reports, there was limited evidence on local data use for routine district level planning or problem solving. Moreover no evidence of stakeholder engagement was found in the decision making process [15]. Figure 1 shows the number of district health office and staff in intervention and control arms.

### Study setting

The study included all districts of North Shewa Zone by allocating 12 of them to the intervention and 12 to the control arm. Pairs of districts were matched based on criteria of designated performance level, distance from zonal capital and presence of Transform Primary Health Care Units (these units had support from a non-governmental organization on data use).One district in each pair was randomly allocated to the intervention arm.

### Sample size

A minimum of 5 (m) health management staff per district health office were included in the sample (120 study subjects in total) was estimated to have 80% power(Z_β_) to detect a difference from 50% (π_0_) of at least 23% points as statistically significant at the 5% level (Z_α/2_) with an intra-class correlation (ICC) of 0.03 (k_1_ = k_0_) using the formula [16].

$$c=1+{({z}_{\alpha /2}+{z}_{\beta })}^{2}\frac{{\pi }_{0}(1-{\pi }_{0})/m+{\pi }_{1}(1-{\pi }_{1})/m+({k}_{0}^{2}{\pi }_{0}^{2}+{k}_{1}^{2}{\pi }_{1}^{2})}{{({\pi }_{0}-{\pi }_{1})}^{2}}$$

### Study participants

The study population included all district health office staff, and study participants included District Health Office heads, reproductive and maternal health officers, child and nutrition officer, Monitoring and Evaluation case team leads and Health Information Technicians. Information was collected from these selected staff at baseline and end line.

Further details on the DIPH intervention strategy and trial method is reported elsewhere [14].

### Study variables

We estimated decision-making culture using raw data on a Likert scale type which ranged from 1 (strongly disagree) to 5 (strongly agree). Using the Likert scale data, we calculated six composite indices each reflecting one aspect of decision-making culture, in each case using the percentile mean index: evidence-based decision making, emphasis on data quality, use of information, problem solving, responsibility and motivation. The study questionnaire was adapted from the PRISM framework [17]. To test the adapted questionnaire and identify any issues with the question sequences and relevance, a pretest took place from June 11–19, 2019. Pretest participants were 13 health facility focal person/unit heads from Bishoftu town district health office. The variable categorization framework is shown in Panel 1below, with details included in Annex 1.

### Data analysis

For all items shown in the operational definition tables (Annex 1), the original Likert scale rating for each item was first converted from 1-to-5 to 0-to-4. The mean score was then calculated by considering all items in each domain and a percentile mean index calculated which ranged from 0 to 100. For instance, for the evidence-based decision-making domain there were 10 items, so to get the corresponding percentile the rate given by the given respondent was summed and divided by 40 (10 items x 4) then multiplied by 100. To get the overall summary measure of decision making culture, the six domain-specific indices for each respondent were summed and divided by six.

For each of the six domains of data use (evidence-based decision making, emphasis on data quality, use of information, problem solving, responsibility and motivation), a percentile mean index and standard deviation was calculated separately for intervention and control districts at baseline and end line. Based on these percentile mean indices the difference in difference estimates with corresponding confidence interval were calculated to estimate the net effect of DIPH implementation. These estimates were adjusted by the study cluster and respondents’ age, gender and level of education by considering these as covariates. This analysis assessed change pre- to post-DIPH intervention, and then compared it between the two groups. This approach eliminates biases resulting from trends in the outcome due to causes other than the intervention. To further avoid potential bias, the end line survey was carried out by an independent data collection team.

Within intervention districts, we assessed differences in perceived utilization among types of staff designation, comparing the district health office head with health information technicians and program officers, using a univariate general linear model or one way analysis of variance (ANOVA).

We considered comparisons between groups as statistically significant using a 5% level. Both SPSS and STATA software were used for analysis.

### Ethical approval

Ethiopian Public Health Institute and London School of Hygiene and Tropical Medicine Institutional Review Board approved this study (Reference Number EPHI 6–13/194 and LSHTM 17,578).

## Results

In this section, we present descriptive information about the study population and data completeness, then show the effects of DIPH intervention on the six domains of decision-making culture through a difference-in-difference analysis (intervention versus control) by adjusting for study cluster; respondents’ age, education and gender for all evidence based decision making, emphasis on data quality, use of information, problem solving, responsibility and motivation. We then show the effect of the DIPH intervention on the overall summary measure of decision-making culture, combining all the six domains. Because there was no observed evidence of confounding factors, we present only unadjusted results. Lastly, we turn to the effect of staff role (district health office head, health information technician and program officers) on the same six domains of decision-making culture, restricting this analysis to intervention districts only.

Both the baseline and end line data collection included 60 respondents from intervention as well as control arms each, five participants per district health office. From 60 respondents in the intervention arm at baseline, 83% were males and in control arm at baseline 87% were males. At baseline, in both intervention and control arms, the mean respondent age was 33 years.

Table 1 shows the net effect of DIPH implementation on decision-making culture at district level. For the domain of evidence-based decision-making, the mean index increased in intervention districts from 55% at baseline to 63% at end line. However, in control districts it decreased from 60 to 57%. Thus, the net effect of the DIPH intervention was an 11 (95% C.I: 6, 15) percentage point increase for perceived evidence-based decision-making.

For the decision-making culture domain of use of information, in intervention districts the mean index increased from 61% at baseline to 81% at end line, while in control districts there was no change between baseline and end line. Thus, the net effect of the DIPH intervention on decision-making culture related to the use of information was a 20 (95% C.I:12, 28) percentage-point increase.

For the decision-making culture domains of emphasis on data quality, problem-solving, responsibility, there was consistent evidence of a positive effect of the DIPH intervention of at least 10% points in the mean index: 16 (95% C.I:8,24) percentage points, 21 (95% C.I:13,29) percentage points, and 10 (95% C.I:4,16) percentage points, respectively.

However, for the decision-making culture domain of motivation, there was no evidence of a positive effect of the DIPH intervention (2% points, 95% C.I: -4, 8).

The overall composite mean index of decision making culture increased in intervention districts from 63% at baseline to 74% at end line, with a net positive effect of the DIPH intervention of 13% points (95% C.I:9,18).

**Table 1:** Estimated net effect of DIPH implementation on decision-making culture at district health office

Decision making domain	Control districts				Intervention districts				DID^2^ Net Effect (95% C.I)^3^	P-Value
	Baseline		End line		Baseline		End line			
	Mean (SD)^1^	N	Mean (SD)	N	Mean (SD)	N	Mean (SD)	N		
Evidence Based Decision Making	60(10)	60	57(8)	60	55(9)	60	63(10)	60	11(7,15)	< 0.001*
Emphasis on Data Quality	70(17)	60	68(19)	60	67(16)	60	81(11)	60	16 (8, 24)	< 0.001*
Use of Information	67(18)	60	67(18)	60	61(17)	60	81(10)	60	20 (12, 28)	< 0.001*
Problem Solving	65(16)	60	58(16)	60	59(14)	60	73(12)	60	21 (13, 29)	< 0.001*
Responsibility	73(13)	60	68(13)	60	70(11)	60	75(8)	60	10 (4, 16)	0.001*
Motivation	64(11)	60	67(12)	60	66(11)	60	71(10)	60	2 (-4, 8)	0.52
Overall decision making culture (composite)	66(10)	60	64(10)	60	63(8)	60	74(6)	60	13 (9, 18)	< 0.001*

* Statistically significant at 5% level

^1^ SD = Standard Deviation

^2^ DID = Difference-in-Difference

^3^ C. I = Confidence Interval

Table 2 shows the differences among district health office head, health information technician, and program officers on the six domains of decision-making culture, in intervention districts at end line, adjusting for study cluster, and respondent age, sex and education.

For decision-making culture related to problem-solving, we found that evidence of a difference between the district health office head, health information technicians and program officers, with the mean index at 68%, 65% and 66%, respectively. The difference between these three health cadres was marginally statistically significant (*p* = 0.053).

Similarly for decision-making culture related to responsibility, we found that evidence of a difference between the district health office head (75%), health information technicians (69%) and program officers (72%), with the difference between these cadres being statistically significant (*p* = 0.009).

At 5% level of significance in intervention districts after controlling for respondent age, sex and education, the differences in the percentile means index among heads, HITs and program officers on evidence-based decision making, emphasis on data quality, use of information, motivation and overall composite mean index were not statistically significant.

**Table 2:** Adjusted mean and analysis of variance for percentile index related DHIS-2 data utilization by staff designation type at intervention districts health office of end line after controlling the effect of respondent age, sex and level of education

Decision making domain	Staff Designation			ANOVA^2^				
	Head (*n* = 24)	HIT^1^ (*n* = 24)	Program Officers (*n* = 72)					
	Mean (SD)	Mean (SD)	Mean (SD)	SS^3^	d.f^4^	MS^5^	F^6^	*P*-value
Evidence Based Decision Making	59(9.5)	60(9.7)	59(10.5)	55.1	2	27.6	0.3	0.782
Emphasis on Data Quality	78(14.3)	73(15.2)	73(15.9)	664.4	2	332.2	1.3	0.282
Use of Information	71(19.3)	71(13.4)	71(18.1)	435.6	2	217.8	0.7	0.492
Problem Solving	68(15.5)	65(13.7)	66(15.1)	1261.2	2	630.6	3.1	0.053*
Responsibility	75(9.6)	69 (7)	72(9.8)	833.0	2	416.5	4.9	0.009*
Motivation	70(9.1)	68(11.5)	69(11.5)	92.1	2	46.1	0.4	0.674
Overall decision making culture (composite)	70(7.9)	67(8.7)	68(9.4)	95.1	2	47.6	0.58	0.559

Note: -

* Statistically significant at 5% level

^1^ HIT = Health Information Technicians

^2^ ANOVA = Analysis of Variance

^3^ SS = Sum of Squares

^4^ d.f = degrees of freedom

^5^ MS = Mean Square

^6^ F = Fisher statistical estimate the mean is adjusted by study cluster

## Discussion

### Summary of the Key findings

In this study, we found that there is a strong evidence that DIPH intervention has improved the overall decision-making culture among key district health administration staff. Key domains of this improvement included e evidence-based decision-making, emphasis on data quality, use of information, problem solving, and responsibility. In addition, we found evidence that decision-making culture depended on the roles of staff within the team - responsibility for reviewing data and using it for problem-solving were considerably more evident among district health office heads than other staff in the intervention districts. However, DIPH intervention showed no evidence of difference in the overall decision-making culture by administrator designation type.

Importantly, this indicates that the intervention helped staff of all designation types in their overall decision making culture.

### Comparison of findings

Public health organisations, such as health departments, are professional establishments that bring together experts with management, logistics, financial, information and clinical skills - as well as personalities and behaviours - as a cooperative unit to attain the objective of improving population health [20]. Organisational culture is a driver of change. Based on commonly held convictions, values & beliefs, organisational culture manifests as sharing roles and responsibilities and implementing processes to influence performance. A highlight of the DIPH intervention on district health management was the positive shift in the culture of evidence-based decision-making using DHIS-2 data. It is important to note that this shift was expected immediately after the introduction of the health management information system to Ethiopia in 2008, [18] leading to improvements in the timeliness and quality of local data, but the culture of routine data use for decision-making across the health system was extremely low [13]. A study conducted in Illu Aba Bora Zone, Ethiopia highlighted that in the absence of any effective intervention, only 50% of study participants had reasonable knowledge of and favourable attitudes towards the use of DHIS-2 [21].

In the African context, a health systems intervention implemented in Cote d’Ivoire reported an increase in a district level data-use score from 40 to 70% [22] - similar to the positive changes reported here. However, the study in Cote d’Ivoire involved a broad-spectrum intervention pertaining to the interrelated technical, behavioural and organisational domains based on the Performance of Routine Information System Management (PRISM) framework [17]. In comparison, DIPH was a more focused intervention that specifically targeted district health management’s data-use and decision-making capacities and practices. Hence, it was more feasible to implement within limited resources.

### Possible mechanism of culture changes due to DIPH

DIPH involved a series of four monthly-cycles that were carried out by district health management staff. The mechanisms of change in data-use culture were distributed over a five-steps DIPH cycle. These, steps start with, assessing the district’s current situation by the district management team. This required systematic review of existing data, district health policies & plans, and other contextual information to identify specific evidence-based MNCH priorities that needed to be addressed. Step 2 was about engaging other departments within the district health office as well as other key stakeholders to understand how collective action could help and how to coordinate it. Engaging stakeholders created motivation and encouraged responsibility for actions on specific health themes. Step 3 involved defining the areas to be improved in a cycle. District health managers discussed challenges related to current themes adapted from the World Health Organization’s framework of six health system building blocks (namely: health workforce, financing/resource allocation, service delivery, governance, access to essential medicines, and health information system) [23, 24]. This helped to identify an area where implementing a complete cycle of DIPH could improve outcomes. In step 4, stakeholders discussed actionable solutions to the problems proposed in step 3 and defined data indicators to measure their progress against the action points. They allocated responsibilities of each action point to respective stakeholders and determined timelines for each action to be completed. The fifth and final step involved ‘following up’ on the plan’s implementation. The stakeholders convened four months later and evaluated progress of each action plan based on target, progress and set timeline.This structure use of information enabled them to track their progress in an effective way. The above discussed steps complete one DIPH cycle for a specific theme challenge.

DIPH was embedded within existing monthly performance-monitoring team meetings. Thus, these routine decision-making management forums were made more efficient, obviating the need to reinvent the wheel with additional meetings or data collection.

The organisational culture was, in practice, intrinsically top-down – management goals, decisions and practices filtered down to service providers. The district health management culture potentially influenced the collaborative, evidence-based decision-making of primary care health service providers in providing optimal services to the populations they serve. The behaviour of a district management leader who demonstrated active engagement with their responsibilities and promoted the use of information to solve service delivery problems was, by force of example, likely to promote similar practices in their service-provider workers. It is interesting to note that perceived cultural change was more pronounced in district health office heads than in health information technicians (HIT) and other programme officers in district management. This might be because heads of the district health office chaired the performance monitoring team meetings where the DIPH strategy was embedded, as well as being the focal person of DIPH-related activities [19], overseeing the process of structured decision-making and providing leadership for the regular functioning of DIPH strategy within the district health office [25].

In summary, the DIPH strategy strengthened data-driven decision-making culture among district health management in Ethiopia. By a more robust health management culture, we specifically mean that most management staff members accepted, practiced and followed a code of conduct that was formulated for effective decision-making to achieve an intended function and impact: i.e., efficient and effective health service delivery [26].

### Limitations

Limitations of our study include limited geographical representation, being based on perceptions, and being limited to a relatively small number of participants in each district. With regards to geographic representation, the study was limited to a single zone and the results may not be generalisable to other settings. Although our research included all the 24 districts in North Shewa zone we had a relatively limited number of study participants per district, which is more a reflection of the composition of the management workforce than a missed opportunity to enrol by the research team. Generally, organisational culture research is based on perceptions and we followed the standard tool to assess it. Although behavioural manifestations of culture can be assessed and verified by observation and testing methods, there are hardly any validated tools available for use in the health system of LMICs. Overall, organisational culture is complex, and we assessed it only from the decision-making perspective. Organisational culture is a substantial area of management research and practice. However, culture research in the health management sector in LMICs is generally minimal.

We therefore strongly recommend further research undertakings in this area to understand the various aspects of health management culture on a deeper level.

## Conclusion

A health system can meet the needs of its population only if it has a robust health administration in pace. Using an existing district health administration platform, the DIPH innovation as a health system-strengthening strategy, has improved organisational culture for data use and structured decision-making for problem-solving in district health administration offices in Amhara region, North Shewa Zone, Ethiopia. Further research is recommended to assess and apply the cultural effect of the DIPH intervention on the other health administration levels of the national health systems such as (health facilities. Zonal health Departments and Regional Health Bureau).

### Electronic supplementary material

Below is the link to the electronic supplementary material.

Supplementary Material 1

Supplementary Material 2

Supplementary Material 3

Supplementary Material 4

## Data Availability

Data are available and will be shared.

## Declarations

All participants gave individual written informed consent. During the primary survey data collection, all ethical principles and methods were carried out in accordance with Ethiopian national research ethics guideline.

## Abbreviations

ANOVA: Analysis of Variance
DHIS-2: District Health Information System-2
DIPH: Data Informed Platform for Health
EPHI: Ethiopian Public Health Institute
HMIS: Health Management Information System
LSHTM: London School of Hygiene and Tropical Medicine
MoH: Ministry of Health
WHO: World Health Organization
