# Supplementary material for: Effect of the data-informed platform for health intervention on the culture of data use for decision-making among district health office staff in North Shewa Zone, Ethiopia: a cluster-randomised controlled trial
Source: BMC Med Inform Decis Mak. 2024 Jul 5;24:190. doi: 10.1186/s12911-024-02597-x (PMC11225382; doi:10.1186/s12911-024-02597-x)
Supplement: Supplementary file 1 — Supplementary Material 1 [file 12911_2024_2597_MOESM1_ESM.pdf]

CONSORT 2010 checklist of information to include when reporting a cluster randomized trial

Section/topic and item No.		Standard checklist item	Extension for cluster designs	Page No.
	**Title and abstract**			
1a		Identification as a randomized trial in the Title (Yes)	Identification as a randomized trial in the Title (Yes)	2
1b		Structured summary of trial design, methods, Results, and conclusions (Yes)		2-3
	**Introduction**			
	Background and objectives:			
2a		Scientific background and explanation of rationale (Yes)	Rational for using a cluster design (Yes)	4
2b		Specific objectives or hypotheses(objective)	Whether objectives pertain to the cluster level, the individual participant level or both (cluster level/district)	4
	**Methods**			
	Trial design:			
3a		Description of trial design (such as parallel, factorial) including allocation ratio (Paired matched with Ratio of 1:1)	Definition of cluster and description of how the design features apply to the clusters. (Yes)	5-6
3b		Important changes to methods after trial commencement (such as eligibility criteria), with reason (No change)		
	Participants:			
4a		Eligibility criteria for participants (Yes)	Eligibility criteria for clusters (Yes)	6
4b		Settings and locations where		

		the data were collected (Yes)		6
	Interventions:			
5		The interventions for each group with sufficient details to allow replication, including how and when they were actually administered (Yes)	Whether interventions pertain to the cluster level, the individual participant level, or both (cluster level/district)	5
	Outcomes:			
6a		Completely defined pre specified primary and secondary outcome measures, including how and when they were assessed (NA)	Whether outcome measures pertain to the cluster level, the individual participant level, or both(NA)	
6b		Any changes to trial outcomes after the trial commenced, with reasons (No change)		
	Sample size:			
7a		How sample size was determined(Yes)	Method of calculation, number of cluster (s) (and whether equal or unequal cluster sizes are assumed), cluster size, a coefficient of intra cluster correlation (ICC or k), and an indication of its uncertainty (Yes)	6
7b		When applicable, explanation of any interim analysis and stopping guidelines (NA)		
	**Randomization**			
	Sequence generation			
8a		Method used to generate the random allocation sequence (NA)		
8b		Type of randomization; details of any restriction (such as blocking, Details		

		of stratification matching if used and block size) (Matching)		6
	Allocation concealment mechanism:			
9		Mechanism used to implement the random allocation sequence (such as sequentially numbered containers), describing any steps taken to conceal the sequence until interventions were assigned (NA)	Specification that allocation was based on clusters rather than individuals and whether allocation concealment (if any) was at the cluster level, the individual participant level, or both (NA)	
	Implementation:			
10		Who generated the random allocation sequence, who enrolled participants, and who assigned participants to interventions (NA)		
10a			Who generated the random allocation sequence, who enrolled clusters to interventions (NA)	
10b			Mechanism by which individual participants were included in clusters for the purposes of the trial (such as complete enumeration, random sampling)(Yes).	6
10c			From whom consent was sought (representatives of the cluster, or individual cluster members, or both) and whether consent was sought before or after randomisation (Yes)	19
	Blinding:			
11a		If done, who was blinded after assignment to intervention (for example,		

		participants, care providers, those assessing outcomes) and how (NA)		
11b		If relevant, description of the similarity of interventions		
	Statistical methods:			
12a		Statistical methods used to compare groups for primary and secondary outcomes(Yes)	How clustering was taken into account (Yes)	7-8
12b		Methods for additional analyses, such as subgroup analyses and adjusted analyses (Adjusted analysis)		8
	**Results**			
	Participant flow (a diagram is strongly recommended):			
13a		For each group, the numbers of participants who were randomly assigned, received intended treatment, and were analysed for the primary outcome (Yes)	For each group, the numbers of clusters that were randomly assigned, received intended treatment, and were analysed for the primary outcome (Yes)	5
13b		For each group, losses and exclusions after randomisation, together with reasons (None)	For each group, losses and exclusions for both clusters and individual cluster members (None)	
	Recruitment:			
14a		Dates defining the periods of recruitment and follow-up (Yes)		
14b		Why the trial ended or was stopped (ended as planned)		
	Baseline data:			
15		A table showing baseline demographic and clinical characteristics for each group (Yes)	Baseline characteristics for the individual and cluster levels as applicable for each group (Yes)	11
	Numbers analysed:			
16		For each group, number of	For each group, number of	

		participants (denominator) included in each analysis and whether the analysis was by original assigned groups (Yes)	clusters included in each analysis (Yes)	5
	Outcomes and estimation:			
17a		For each primary and secondary outcome, results for each group, and the estimated effect size and its precision (such as 95% confidence interval) (Yes, DID estimate with 95% C.I)	Results at the individual or cluster level as applicable and a coefficient of intra cluster correlation (ICC or k) for each primary outcome (Yes, k or cluster adjustment)	11-12
17b		For binary outcomes, presentation of both absolute and relative effect size recommended (NA)		
	Ancillary analysis:			
18		Results of any other analyses performed, including subgroup analyses and adjusted analyses, distinguishing pre specified from exploratory (Yes, adjusted analysis)		9-10
	Harms:			
19		All important harms or unintended effects in each group (NA)		
	**Discussion**			
	Limitations:			
20		Trial limitations, addressing sources of potential bias, imprecision, and, if relevant, multiplicity of analyses (Generalisability)		17
	Generalisability:			
21		Generalisability (external validity, applicability) of	Generalisability to clusters and/or individual	

		the trial findings (NA)	participants (as relevant) (NA)	
	Interpretation:			
22		Interpretation consistent with results, balancing benefits and harms, and considering other relevant evidence (NA)		
	**Other information**			
	Registration:			
23		Registration number and name of trial registry (ClinicalTrials.gov ID: NCT05310682, Dated 25/03/ 2022)		79-80
	Protocol:			
24		Where the full trial protocol can be accessed, if available(Attached in separate file)		
	Funding:			
25		Sources of funding and other support (such as supply of drugs), role of funders (The study was funded by Bill and Melinda Gates Foundation, but no other support (drugs) from funder)		20
