# Supplementary material for: Effect of the data-informed platform for health intervention on the culture of data use for decision-making among district health office staff in North Shewa Zone, Ethiopia: a cluster-randomised controlled trial
Source: BMC Med Inform Decis Mak. 2024 Jul 5;24:190. doi: 10.1186/s12911-024-02597-x (PMC11225382; doi:10.1186/s12911-024-02597-x)
Supplement: Supplementary file 2 — Supplementary Material 2 [file 12911_2024_2597_MOESM2_ESM.pdf]

**Annex 1: Operational Definitions**

| **Decision making Domain** | **Definition** |
|---|---|
| *Evidence Based Decision Making* | The extent to which the district health office believes that decision making in the district health system is guided by DHIS-2 data use. |
| *Emphasis on Data Quality* | The extent to which the district health office gives importance to data quality. |
| *Use of Information* | The extent to which district health office staff and supervisors use data from DHIS-2 for setting, planning and monitoring targets; and display data for monitoring set target. |
| *Problem solving* | The extent to which staff and supervisors in district health office believe that health system problems can be solved using DHIS-2 data. |
| *Responsibility* | The extent to which staff in district health office feel responsible to accomplish tasks and commitment using DHIS-2 data. |
| *Motivation* | The extent to which staff in district health office feel enthusiastic about their role and responsibilities related to collecting information needed to monitor performance at different level . |
