# Supplementary material for: Effect of the data-informed platform for health intervention on the culture of data use for decision-making among district health office staff in North Shewa Zone, Ethiopia: a cluster-randomised controlled trial
Source: BMC Med Inform Decis Mak. 2024 Jul 5;24:190. doi: 10.1186/s12911-024-02597-x (PMC11225382; doi:10.1186/s12911-024-02597-x)
Supplement: Supplementary file 3 — Supplementary Material 3 [file 12911_2024_2597_MOESM3_ESM.pdf]

Annex 2. List of variables considered under the six decision making domains

**Domain**	**Items/Variables**
Evidence Based Decision Making	Decision are based on:-<br>o Personal preference of decision makers (Reverse rating)<br>o Supervisor's directives (Reverse rating)<br>o Evidence/fact/data<br>o History (e.g What was done last year)<br>o Funding directives from higher levels<br>o Political consideration (Reverse rating)<br>o Official health sector strategic objective<br>o Locally identified health needs of the population<br>o The relative cost of intervention<br>o Participatory decision making by obtaining input from relevant staff
Emphasis on Data Quality	Supervisors:-<br>o Emphasise that DHIS-2 data quality procedure be followed in the compilation and submission of periodic reports<br>o Conduct DHIS-2 data quality checks at points where data are captured, processed or aggregated<br>o Provide regular feedback on reported DHIS-2 data quality (accuracy of data compilation/reporting) to the staff responsible for compiling and reporting the data.
Use of Information	Supervisors:-<br>o Use DHIS-2 data for service performance monitoring and target setting<br>o Ensure that regular meetings are held where DHIS-2 data and information are discussed, performance reports are presented and reviewed, decisions are made, follow-up actions are identified, and their implementation is monitored<br>Staff:-<br>o Use DHIS-2 data for day-to-day management of the facility and district (e.g service delivery, financial, commodities, and human resource management)<br>o Prepare DHIS-2 data visuals (graphs, tables, maps etc) showing progress toward targets (indicators, geographic and/or temporal trends, or situation data).
Problem solving	Supervisors:-<br>o Emphasize the need to use DHIS-2 data to identify potential gender related disparities in service delivery or use<br>o Use DHIS-2 data to solve common problems in service delivery<br>o Use sex-disaggregated or gender-sensitive DHIS-2 data to identify and/or solve gender related problems in service delivery<br>o Can evaluate whether an intervention achieved the target or goal
Responsibility	Staff:-<br>o Complete DHIS-2 tasks (reporting, processing/aggregation, and/or analysis) in a timely manner (i.e meet appropriate deadlines)<br>o Display commitment to the DHIS-2 mission (i.e to generate and use good quality, accurate complete, and timely data for evidence based decision making)

	o Pursue national targets and set feasible local targets for essential service performance<br>o Feel "personal responsibility" for failing to reach performance targets<br>o Admit mistakes if/when they occur and take corrective action
Motivation	Staff agree with these statements:-<br>o I feel discouraged when the data that I analyzed/present are not used for taking action (either for monitoring or decision making)<br>o I find analyzing and presenting data to be tedious (i.e repetitive or duplicative)<br>o I find that the data that I analyze and present burdens my workload, making it difficult for me to complete my other duties<br>o Analyzing and presenting data is meaningful/useful for me<br>o I feel that the data I analyze and present are important for monitoring the performance of the health services provided at my facility/unit<br>o My work of analyzing and presenting data is appreciated and valued by supervisors<br>o I feel that data analysis and presentation is not the responsibility of healthcare providers
