# Supplementary material for: Effect of the data-informed platform for health intervention on the culture of data use for decision-making among district health office staff in North Shewa Zone, Ethiopia: a cluster-randomised controlled trial
Source: BMC Med Inform Decis Mak. 2024 Jul 5;24:190. doi: 10.1186/s12911-024-02597-x (PMC11225382; doi:10.1186/s12911-024-02597-x)
Supplement: Supplementary file 4 — Supplementary Material 4 [file 12911_2024_2597_MOESM4_ESM.pdf]

"Protocol (File 1): (Data-Informed Platform for Health (DIPH) – health system strengthening innovation in North Shewa Amhara region, Ethiopia)"

ETHIOPIAN PUBLIC HEALTH INSTITUTE
INSTITUTIONAL REVIEW BOARD (EPHI-IRB)
RESEARCH PROPOSAL FORM

Project No.____________________ (To be given by EPHI-IRB)

Data-Informed Platform for Health (DIPH) – *health system strengthening innovation in North Shewa Amhara region, Ethiopia*

Person(s) undertaking the project in order of contribution

**Name**	**Qualification ((Area of study & Degree)**	**Institution/Company**	**Country**
**From London School of Hygiene and Tropical Medicine**			
Bilal Avan	MD PhD	London School of Hygiene and Tropical Medicine	U.K.
Joanna Schellenberg	PhD	London School of Hygiene and Tropical Medicine	U.K.
Mehret Dubale	MSc	London School of Hygiene and Tropical Medicine	Ethiopia
Della Berhanu	PhD	London School of Hygiene and Tropical Medicine	Ethiopia
Lars Åke Persson	MD, PhD	London School of Hygiene and Tropical Medicine	Ethiopia
**From Ethiopian Public Health Institute**			
Girum Taye	MSc	Ethiopian Public Health Institute (EPHI)	Ethiopia
Abebe Bekele	MSc	Ethiopian Public Health Institute (EPHI)	Ethiopia

**Source of budget: Bill and Melinda Gates Foundation through the London School of Hygiene and Tropical Medicine**	**Total budget required: ETB 10,528,180.01 (USD 396,028.)**

Contact address of the PI: Tel. Address +442079272317 Email: Bilal.Avan@lshtm.ac.uk

Contact address of first co-investigator: Tel. Address +251932223164 Email: girumt2000@yahoo.com

**Study period: 30 months**	**Date of commencement: August 2019**	**Date of completion: July 2021**

Institution at which study to be conducted: Ethiopian Public Health Institute

Table of contents

Abbreviations and acronyms 3

List of appendices 4

Summary 5

Background and justification 7

Objectives of DIPH project 14

Materials and methods 14

Strengths and limitations of the DIPH project 18

Communication and dissemination of study findings 18

Ethical considerations and review process 18

Implementation of DIPH project 19

Cost of DIPH project, budget breakdown 19

Benefits of the Study's Results 22

Research Collaborators 22

Assumptions, risks and mitigations 23

Facilities available for the study 23

Authorship right 23

Declaration of conflict of interest 23

References 24

Assurance of the Principal Investigator: 25

Appendices 27

1. Facilitator ID 37

3. Data sources 39

References 54

Abbreviations and acronyms

DIPH: Data-Informed Platform for Health

EPHI: Ethiopian Public Health Institute

LSHTM: London School of Hygiene and Tropical Medicine

IDEAS: Informed Decisions for Actions in Maternal and Newborn Health

FMoH: Federal Ministry of Health

HMIS: Health Management Information System

DHIS2: District Health Information System 2

PI: Principal Investigator

List of appendices

Supplementary Annex (A): Map of North Shewa within broader Amahara region. (and if possible map of woredas within north Shoa zone)

Supplementary Annex (B): List woreda by type: and population size in North Shoa zone

Supplementary Annex (C): Health Profile of North Shewa zone on selected Maternal and Child Health indicators

Supplementary Annex (D): information about the study

Supplementary Annex (E): DIPH Study Instruments

Summary

Appraisal and use of local data for health system planning and decision-making are often limited, despite advances in data-gathering operations in lower- and middle-income countries. A data-sharing culture and opportunities for collaborative action-planning among health-stakeholders are lacking. Local health administrators in such settings often have limited capacity to analyze and use data for decision-making.

The overall aim of the Data-Informed Platform for Health (DIPH) is to improve Maternal, New-born and Child Health (MNCH) programmes and services at the Woreda level. The DIPH strategy does this by bringing together data on inputs and processes in order to promote the use of local data for decision-making, priority-setting and planning through the introduction of a structured decision-making process at the Woreda level. This resonates closely with the Ethiopian Health Sector Transformation Plan in 2015 [ref], which identifies the information revolution as part of the transformation agenda. Promoting a culture of information-use at, or close to, the point of collection is central to the information revolution.

The transition of the DIPH from a concept to a health-systems initiative is based on a series of implementation-research activities, including a background assessment and the development and testing of the intervention. Our formative research showed that there is only a limited structured district decision-making process, with limited interactions between departments and little formal Woreda-level data-sharing. The DIPH is embedded in existing Woreda decision-making approaches - e.g. Performance Review Teams at Woreda level - adding a structured process of coordination between different departments, and formal data-sharing for evidence-based decision-making, planning and resource allocation according to local health priorities.

Conceptually, the DIPH strategy uses a structured set of processes involving five pre-defined steps, and standardized job-aids corresponding to each step, to facilitate linking data from health and associated departments and stakeholders. A typical DIPH cycle has five steps around a health theme, which take about three to four months to complete. Technical assistance is provided by the induction, orientation and handholding of the Woreda stakeholders during the implementation of the initial cycles.

The DIPH job-aids – a set of standardized job-aids (paper forms or web-based interface) corresponding to each of the five steps – are designed to help in the organization and interpretation of data from multiple sectors involved in the delivery of services around the chosen theme using a common data-sharing platform. They areaimedat Woreda leadership and management teams using, inputting and processing data systematically for decision-making, planning and progress-monitoring of the theme.

In Ethiopia, action-research will be employed to adapt, implement and evaluate the DIPH approach in a series of overlapping phases.

1. **Developmental phase (August 2018 – May 2019):** Engage with Ethiopian Federal Ministry of Health and synchronize with similar projects, Adapt DIPH tools and strategies for the local context, and Finalize the selection study area. This phase has been successfully completed, and we anticipate that the study will be implemented in North Shewa zone of Amhara region (12 DIPH, 12 Non-DIPH Woredas).

2. **Implementation phase (August 2019 - July 2021): Implementation for 24 months (two fiscal years:** first year for embedding, and second year to establish effectiveness), up to six four-monthly cycles conducted in each of 12 Woredas.

3. **Evaluation phase (2019 - 2021):** *Process evaluation* to understand and improve on-going implementation issues. *Before-and-after comparison* of intermediate outcomes in intervention and comparison areas via health facility and Woreda surveys.

Budget: The estimated cost of DIPH implementation and evaluation will be *will be 10,528,180.01 Ethiopian birr.*

This study is a collaboration between the Ethiopian Public Health Institute (EPHI) and the London School of Hygiene and Tropical Medicine (LSHTM) under the remit of the IDEAS (Informed Decisions for Actions in Maternal and Newborn Health) project. In Ethiopia, we anticipate federal government interest in the findings of the DIPH approach, given its potential to strengthen district level health systems by enabling data-driven decision-making, which could be instrumental in operationalizing the Ethiopian Health Sector Transformation Plan.

Background and justification

In low-resource settings, the use of local health data for planning is usually limited. It is typical for diverse health-providers – relevant local government departments, NGOs, private providers – to work in parallel with no formal mechanism to share their data or plan their services collaboratively. If mechanisms for data-sharing were improved, there would be opportunities to reduce duplication, make better use of resources and meet community health needs more fully.

In Ethiopia, multiple sources of data exist at the district level: the Health Management Information System (HMIS) reflects health facility utilisation and performance; programme staff report on human and physical resources; relevant government departments (e.g. agriculture) have information on nutrition, social welfare and food security; and non-governmental organisations report on community-based activities. Access to shared data could empower local decision-makers to make better decisions, derived from a broader base of evidence, and deliver health services on the basis of available resources.

The initial phase was started with the DIPH co-creation workshop in Addis Ababa, held on 25 April 2018 and chaired by the Federal Ministry of Health's (FMOH) Policy and Planning Department (PPD). Participants included representatives from: the Ethiopian Public Health Institute, Ethiopia Data Use Partnership - DUP, Last 10 Kilometres, and the Bill and Melinda Gates Foundation. The objective of the workshop was to collaboratively develop a plan to: a) adapt DIPH and its implementation through research activities relevant for Ethiopian health systems; and b) identify and establish linkages with complementary initiatives supporting the use of local data.

Based on the recommendations of the workshop, the objectives of the developmental phase were formulated:

1. To understand how routine data (DHIS, LMIS, PFSA and any other data collected on an ongoing basis) are used at present at Woreda level for problem-solving.
2. To understand what are the existing decision-making and/or data-review forums in Woredas.
3. To determine the short-term needs (if any) of Woreda health-system managers in terms of being able to move towards a more structured approach to problem-solving such as DIPH.
4. To introduce respondents to DIPH's process-steps and interviews, and record their views on its feasibility.

In order to operationalize these objectives, a series of exploratory activities was carried out, including consultation with federal, regional and Woreda-level health managers, direct observation of key problem-solving forums/meetings, and workshops with district-health management staff (Appendix: information-collection instruments).

Guided by the findings of the development phase,

1. We adapted DIPH tools and strategies from West Bengal to the Ethiopian context, and
2. A DIPH training curriculum was developed. The overall aim of this curriculum, which is the basis of a training handbook, is to provide tools and knowledge for improved decision-making at the district level, utilizing available data. Its target audience are district health officers, in both administrative and managerial roles within health systems. The first half of the course will cover the four core skills of decision-making,

stakeholder-engagement, data-use, and monitoring health-system performance. Next, participants will be provided with an overview of district-level organizational structure, followed by an introduction to DIPH, steps in the DIPH cycle, primary and supplementary forms, roles and responsibilities, DIPH meetings, and, finally, how to use the DIPH interface.

Core Concept

The overall aim of the Data-Informed Platform for Health - or DIPH - is to improve MNCH programmes and services through data-driven decision-making at the district level. It is a strategy for strengthening health systems. The DIPH does this by bringing together a variety of stakeholders into a single platform, and facilitating their understanding and use of key district-level data.

Our focus is MNCH, although the DIPH concept has broad applicability. The primary objectives of the DIPH are to promote the use of local data from programmatic activities for: 1) decision-making, priority-setting and planning at the district health administration level; and 2) appraisal of health services and programmes.

A district is considered to be the operating unit for the DIPH based on the assumption that this is the lowest effective administrative level of decision-making in a health system in Ethiopia. The DIPH concept has its roots in the 'District Evaluation Platform approach1, 2, 3.

The DIPH approach will bring governmental and non-governmental service providers to a common forum on a regular basis, in order to share data according to an agreed plan and to use the resulting information as a tool in priority-setting for resource allocation and needs-assessment for the further acquisition of funds.

The DIPH does not try to reinvent the wheel - it is embedded in decision-making procedures that already exist at the district level. What DIPH adds is a structured process of coordination between government and private stakeholders of formal data-sharing for evidence-based decision-making, planning and resource allocation. Moreover, the DIPH is conducted in accordance with local health priorities.

Problem statement

Decision-making within a health system requires stakeholders to reach consensus on a particular health theme – e.g. antenatal care - in order to address challenges in the management and delivery of health services.

When making decisions about issues of a health theme at the local level, using data is instrumental in setting priorities, allocating resources, effectively monitoring services and making realistic plans. However, a) data captured by different stakeholders – health and non-health departments, non-governmental organizations, or the private sector – need to be brought to a common platform to enable evidence-based decision-making, b) in countries and regions of limited resources and expertise, the possibilities of using local data for health system planning and decision-making are often limited, c) the data that is available may be of poor quality, and a culture of data-sharing may not have developed, and d) when decisions are made about health service management, those making the decision often have a limited capacity to understand and use the data available to them.

The DIPH brings together key data from the district level and promotes the use of local data for decision-making, priority-setting, planning and course-correction at the district level. This is done through the introduction of a structured and collaborative decision-making process. Further details of the underlying core principles of the DIPH strategy are described in figure 1. As such, while the DIPH approach has been applied primarily to Maternal, Newborn and Child Health, it can be applied to any area of healthcare.

How does the DIPH work?

The DIPH is delivered as a package of job aids and guidelines. Structurally, the package is made up of three main elements: **Firstly,** the package involves the grouping of stakeholders who are brought together to deliberate on issues in a virtual platform facilitated by regular meetings. The membership of this virtual platform is flexible and responds to the needs of the issues in focus. **Secondly,** the DIPH package facilitates these stakeholder meetings in ***five steps*:** Assessment, Engagement, Definition, Planning and Follow-up. These five steps together make up one whole cycle of the DIPH and, in practice, take 3-4 months to complete. Each cycle looks at a specific health theme, identified in the early stages of the cycle itself. **Thirdly,** the DIPH package also includes a digital interface where everyone involved in the process can regularly review data and check on progress.

Supplementary Figure (1): Core Principles of the DIPH strategy

**1**	**Integrated support of health systems**
	The primary focus is on strengthening health systems through effective decision-making and collaboration by converging across health and social sectors.
**2**	**Data driven**
	Locally-generated data is central to decision-making and management at the Woreda level.
**3**	**Health systems performance**
	Problem-solving is by employing the WHO health system blocks framework, for health system management and monitoring performance at the district level.
**4**	**Embeddedness**
	The Embeddedness of the DIPH into existing decision-making at the district and above levels with respect to<br>1. Participatory decision-making<br>2. Government-specified priorities
**5**	**Responsiveness**
	Identification of a thematic focus in response to local needs at the district level, and execution of the implementation plan to strengthen health systems.
**6**	**Implementation support**
	Providing assistance to district administration staff in terms of training and capacity-building:<br>1. Inter-sectoral collaboration 3. Data use and<br>2. structured decision-making 4. Monitoring health systems performance
**7**	**Outcome focused**
	Improving health data-management, data-use and effective decision-making culture at the district level.

Development of DIPH

Until now, the DIPH development has been led by the IDEAS project. In working to transform the DIPH from a concept to a health systems initiative, we undertook a series of implementation research activities.
This included conceptualization, feasibility assessment, and the development and testing of the DIPH strategy. The feasibility of establishing the DIPH was assessed in the context of district health systems in various countries. The feasibility of the DIPH was assessed in five dimensions: technology and systems, economics, law and politics, operations, and schedule feasibility. Through this assessment the primary structure and content of the DIPH evolved. In the Health Policy and Planning Journal we reported on the feasibility of establishing the DIPH in the context of district health systems in India, Nigeria and Ethiopia, taking into consideration five dimensions: technology and systems, economics, law and politics, operations, and schedule feasibility4, 5, 6, 7, 8. Our papers describe the potential of health data from multiple sources. If utilised regularly and in a more structured way, they would greatly enhance evidence-based decision-making.

Operationalization of the DIPH concept

Figure 2 represents the sequence and links between the 5 steps of DIPH. Usually, steps 1, 2 and 3 comprise a core team of district-level officials, including District Programme-Managers, Heads of Department, and Statistical and Research Officers of health and non-health departments. However, more stakeholders can

be included - according to the nature of the steps and the health challenge in focus during the cycle. This includes health and non-health departments in the public sector along with private service-providers and NGOs.

Supplementary Figure (2): Five-step DIPH cycle

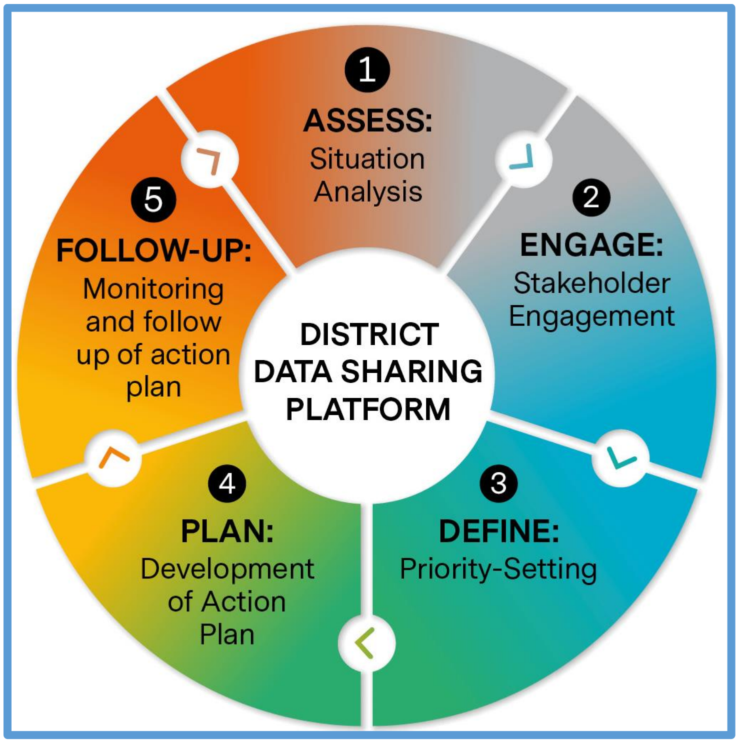

Step 1 – **'Assessing'** the current situation of the district
The first step of structured decision-making requires understanding the existing situation in the area of interest, e.g. Maternal, Newborn and Child Health. Understanding the situation requires a systematic review of existing of data, district health policies and plans - as well as other contextual information – in order to identify the specific health theme that needs to be addressed. This step is also marked by the selection, by group consensus, of the theme leader, who is responsible for coordinating the progress of the DIPH cycle. This process establishes a detailed and realistic picture of the coverage of services, and what human, material and financial resources are available in a particular district.

Step 2 is about engaging other departments and key people to understand how collective action can help, and how to coordinate it. Engaging stakeholders generates ownership of actions on specific health themes. The process will enhance engagement of, and collaboration among, stakeholders. It involves the same members as in step 1.

Step 3 involves **'Defining'** the areas to be improved in a cycle. Usually in this third step, the core team are joined by a wider group of stakeholders relevant to the selected theme. Everyone involved in the DIPH further defines the challenges related to the selected theme in line with the World Health Organization's *health system 6 building blocks**. This then helps identify an area where implementing a full

cycle of the DIPH can potentially help improve outcomes. Steps one, two and three can potentially be conducted in one day.

In step 4, which can be actioned immediately after step three, stakeholders are joined by a senior representative of the district administration. The stakeholders discuss actionable solutions proposed in step 3, and define indicators to measure their progress against the action-points. They allocate the responsibilities of each action point to the concerned stakeholders and define a timeline for it to be completed.

The fifth and final step involves "Following up" on the implementation of the plan set out in step 4. The DIPH cycle theme leader monitors the implementation of the plan by all the stakeholders over a period of three to four months and provides support wherever needed.

In step 5 all DIPH stakeholders from step 4 meet again, three to four months later. The group evaluate the action plan based on the target and timeline developed, and this enables them to track progress. If the action point is not on track, they can review and redefine the responsibilities and timeline. During the follow-up period, the theme leader of the cycle receives monthly progress reports from the sub-district level. This completes a full cycle of the Data-Informed Platform for Health for a specific theme-related challenge in Maternal and Newborn health, or any other health theme.

During all five phases of the Data-Informed Platform for Health, stakeholders use a variety of resources developed to support all the steps of the cycle. These resources bring structure to the discussions, and help capture data and feed it efficiently into the next step of the cycle.

A guide to all the resources available in the DIPH package - including the DIPH web interface - can be found at ideas.lshtm.ac.uk

DIPH was developed and successfully tested in West Bengal over the course of two years. External evaluation suggested the DIPH prototype phase was successful in achieving its goals. It also facilitated further development of the DIPH Programme Theory which has been outlined in figure 3, i.e. mechanisms, context, and determinants of data-sharing and data-use for decision-making, planning, progress-monitoring and follow-up among inter-sectoral stakeholders using DIPH9 .

Supplementary Figure (3): DIPH Programme Theory

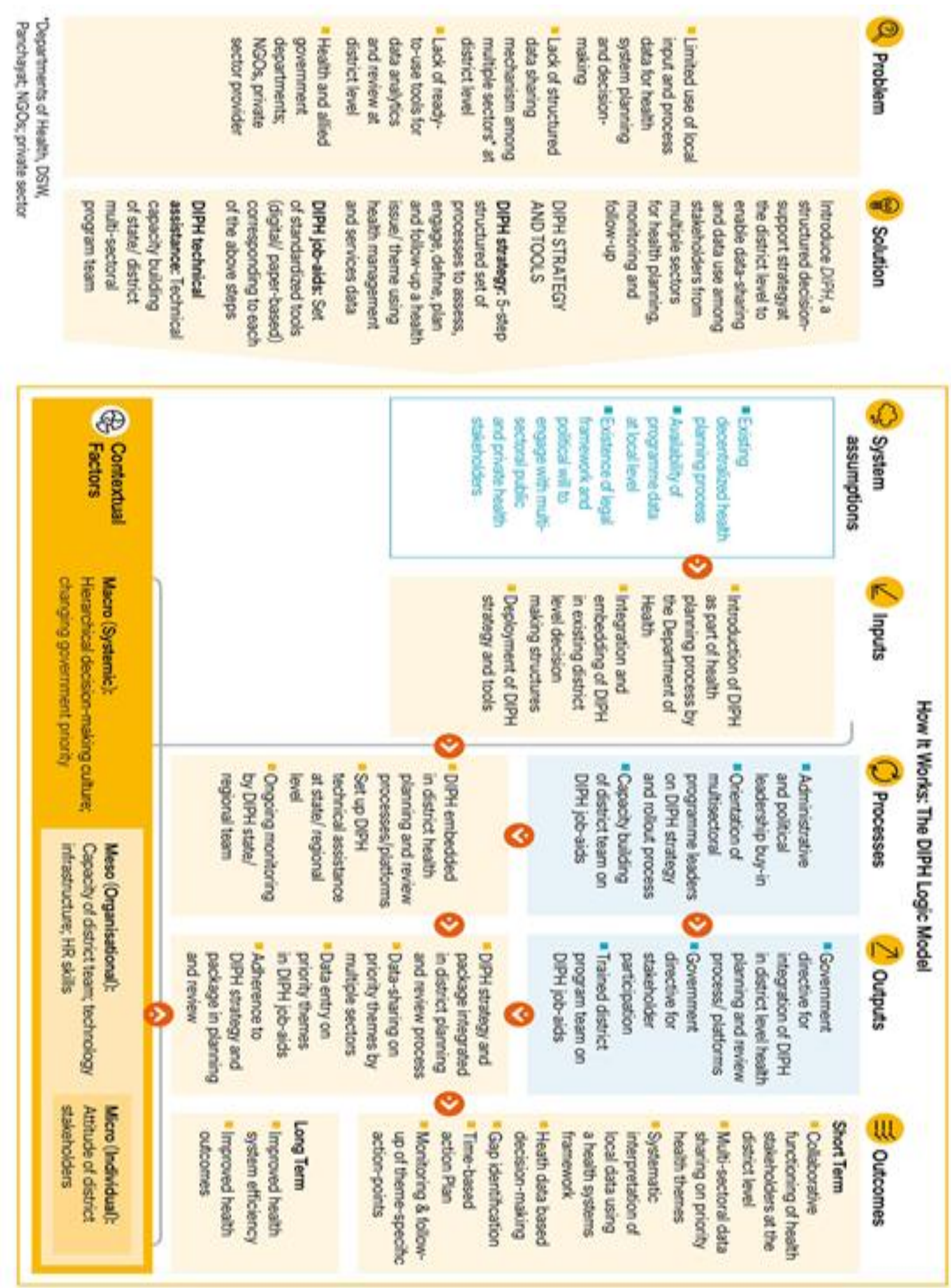

Objectives of DIPH project

Overall aim:

We propose a project of implementation research with the aim to enhance interaction among district-level health personnel and linkage of databases to improve coordinated decision-making and planning by strengthening health systems through capacity-building and effective use of data for decision-making.

Specific research objectives:

1. To systematically strengthen the Woreda health system using the DIPH strategy by developing a coordinated decision-making and planning through the Data-Informed Platformfor Health strategy
2. To evaluate the effectiveness of embedding the Data-Informed Platform for Health within the Woreda health system with respect to improvement in Woreda health performance in terms of data management and data-driven decision-making as compared to Non-DIPH woredas.

Materials and methods

We propose an action-research approach to adapt, implement and evaluate the DIPH approach in Ethiopia. We aim to work in close collaboration with national and local institutions, and engage key government stakeholders. This will provide essential information for policy-makers and decision-makers about the usefulness of DIPH.

The total duration of this work will be three years which is going to be implemented in phases.

Study Design

A quasi-experimental study with pre and post comparisons will be applied. Study woredas will be considered as clusters and equal number will be allocated for presence and absence of intervention arm. The study will be conducted in all Woredas of North Shewa Zone by allocating 12 of them in intervention and 12 in non-intervention arms. A matched peer random allocation will be employed. To reduce subjective allocation, matching will be done based on Woredas' performance level and distance.

Study area: based on the criteria of a) receptiveness of the zonal health office, B) manageable distance from Addis Ababa, the Amhara region was shortlisted for the implementation phase of DIPH.

Amhara regional state consists of 10 administrative zones, one special zone, 105 Woredas, and 75 urban centres. Amharic is the working language of the state. The capital of the region is Bahir Dar, where Ethiopia's largest inland body of water, Lake Tana, is located. The state of Amhara covers an estimated area of 170,752 square kilometres, and its projected population for 2019 G.C. is 21,844,000.

The study will be implemented in North Shewa, one of the administrative zones in the Amhara region. Geographically, it is bordered in the south and west by the Oromia region, in the north by South Wollo, in the north-east by the Oromia Zone, and in the east by the Afar Region. The total area is 15,936.13 square km, with a population density of 115.30. According to the 2007 census conducted by the Central Statistical Agency of Ethiopia (CSA), this zone has a total population of 1,837,490. 11.66% are urban dwellers. Towns and cities in North Shewa include Ankober, Debre Berhan and Shewa Robit. Amharic is the first language of 92.97% of the population, followed by Oromiffa (6.32%). 94.71% of the population are Ethiopian Orthodox

Christians. Details of selected maternal and child-health indictors and health-system infrastructure in the proposed administrative zone are given in Annex I, II and III.

This development phase has been successfully completed, and we anticipate the study will be implemented in all 24 Woredas of the North Shewa zone (i.e. 12 DIPH, 12 non-DIPH).

Implementation phase (August 2019-July 2021): up to six four-monthly cycles of DIPH each will be conducted in the 12 woredas of North Shewa zone over 24 months (i.e. two fiscal years: the first year to complete the embedding of the strategy within the system, and the second year to establish effectiveness).

The Data-Informed Platform for Health will be implemented in the North Shewa administrative zone of the Amhara region. Its Woredas will be stratified by urban and rural, and 12 intervention and 12 comparison Woredas will be randomly selected in collaboration with the zonal health department.

Embedding: The Performance Review Team meetings of the district health administration, which is entrusted with integrating planning and facilitating data-driven and collaborative decision-making, will be used as a platform to introduce the DIPH. Activities will focus primarily on:

1. Strengthening the Performance Review Team forum for the engagement of all government and non-governmental stakeholders in order to identify challenges and solutions, and to assess resource allocation and responsibilities. These meetings will be convened with the aim of consensus-building and collective decision-making to improve health systems and services in their areas.
2. Promoting the critical review and regular use of local data to understand health-system progress at the Woreda level.

Training: Prior to the initiation of the first DIPH cycle, district health management staff of DIPH Woredas will be provided with three days' intensive residential training based on the specifically-designed curriculum (described above) in their zonal city. On average, six staff members per Woreda will be trained by the research team. Along with the Woreda's health department head, programme officers and an HMIS focal person, experts from the zonal health department will be included in the training. The three-day residential training will be conducted by the DIPH research investigators team

Field research team: Overall, the field team will comprise one DIPH regional coordinator, four DIPH support supervisors and one data manager. The positions of DIPH support supervisor will be filled by applicants who have completed their MPH with a minimum of two years' experience in the Ethiopian health system, while the regional coordinator will have at least 10 years' experience of research coordination. The data manager should have an MSc. in computer programming. Recruitment will be carried out through EPHI in collaboration with IDEAS-LSHTM.

Each support supervisor will be assigned three Woredas where they will provide overall technical support in implementing the DIPH strategy. This will consist of inducting, orientating and handholding Woreda stakeholders during the implementation of the initial cycles, participating in Woredas' monthly performance-review meetings, and monitoring collected data. Data-collection will be performed on tablets using appropriate software for electronic data collection. Data collected from the field will be sent to EPHI's server

on a daily basis through the Internet File Streaming System, a technology for transferring data already installed at EPHI.

Evaluation phase (2019-2021): This will have three aspects:

I. **Process evaluation** – to understand and address on-going implementation issues. The monitoring of DIPH in-progress will address the following questions:

1. To what extent is health data used for problem-solving and delivering health services in each DIPH cycle?
2. What is the level of engagement of various health stakeholders?
3. What are the key mechanisms for data-sharing and consensus-building among the range of health-service stakeholders at the Woreda level?
4. What are the facilitators of, and barriers to, sustaining the DIPH strategy?

Data will be collected on structured forms by DIPH support supervisors for each cycle throughout the implementation of DIPH. (Appendix: DIPH Study Instruments, Section B: monitoring framework, definition and indicators). Synthesised findings will be presented periodically to respective Woreda staff so that learning can used to improve the next cycle.

II. **Before-and-after comparison** of health-system outcomes in intervention- and comparison-district (Woreda) surveys to assess changes resulting from DIPH implementation for at least two fiscal years, in terms of

1. Health Information System performance at the district level (the summary measure will be created based on the constructs of 1) essential infrastructure for data management, 2) data diversity, 3)reporting timelines, 4) data-quality assessment mechanisms, and 5) data use.

2. Governance of data-driven decision-making at the district level (the summary measurewill be created based on the constructs of 1) evidence-based decision-making, 2) participatory decision-making, 3) understanding value of data, 4) health system support for data-use/data-driven decision-making, and 5) accountability).

The summary measures/indices will be calculated for each of the 24 Woredas at baseline and at endline, and will range from 0-100%. They will also be categorized to create indicators to determine proportion change for before and after comparison.

Inclusion criteria

- All Woredas in North Shewa zone will be included in the study.
- All managerial and administrative Woreda staff.

Exclusion criteria

Study participants who do not consent will be excluded.

Timing: the baseline study will be conducted in early August 2019, prior to the DIPH-training of Woreda staff, while the end-line survey will be conducted in late July 2021, after the end of the sixth DIPH cycle.

Study participants: The survey respondents will be the head of the health department, all programme officers and data managers at the district level. All eligible district staff will be interviewed, except those working in their respective districts for less than six months. We anticipate on average 6 -7 eligible staff would be available in each Woreda and we aim to include all of them in the study.

Supplementary Table (1). Sample size for before-vs.-after comparison

Indicator	Expected level at baseline	Expected Minimum percentage point increase	Health management staff assessed/ Woreda	No. of Woredas /study arm
Health Information System performance at the district level	50%	23%	5	12

The sample size estimations for the required number of Woredas required per study arm is based on the statistical formula recommended by Hayes et al10. i.e.

$$c = 1 + (z_{\alpha/2} + z_{\beta})^2 \frac{\pi_0(1-\pi_0)/m + \pi_1(1-\pi_1)/m + (k_0^2\pi_0^2 + k_1^2\pi_1^2)}{(\pi_0-\pi_1)^2}$$

Based on the calculations reported in Table above, a minimum sample size of 60 health management staff per study arm (120 in total) would have 80% power to detect a difference of at least *25 percentage points* as statistically significant.

Where:-
C is number of clusters required (Woredas)
$Z_{\alpha/2}$ is confidence level (95%)
Z_{β} is required power (80%)
π_0 is true proportion in the absence of intervention (50%)
π_1 is true proportion in the presence of intervention (77%)
m is individuals sampled in each cluster/woreda (60)
k_0 is coefficient of variation of true proportion in the absence of intervention (0.03)
k_1 is coefficient of variation of true proportion in the presence of intervention (0.03)

Based on the calculations reported in Table above, a minimum sample size of 60 health management staff per study arm (120 in total) would have 80% power to detect a difference of at least *25 percentage points* as statistically significant.

Data-collection: After a detailed pilot testing (Appendix: DIPH Study Instruments, section B) , baseline survey data-collection will be carried out by DIPH field teams. For end-line survey data-collection, an independent team of four data-collectors and one supervisor will be recruited from the zone for one month. Data-collectors will be recruited by EPHI and will, as a minimum, have a Master's degree. Their classroom training on the survey instruments will be followed by a written assessment on the survey contents. Data-collectors will be provided with a field manual for reference during fieldwork. Job descriptions – with the roles and

responsibilities of each team member, and expected day-to-day deliverables - will be provided. The survey will be piloted to test survey procedures and tools, and a detailed standard operating-procedures document for the survey will be provided to the data-collection team.

Data Analysis

A detailed analysis plan will be prepared and technically reviewed by all research stakeholders prior to the completion of data-collection, answer the extent to which the DIPH has changed Health Information System performance and data-driven decision-making.
An additional outcome of the DIPH evaluation would be the revision of the DIPH programme theory for the Ethiopian context.

Strengths and limitations of the DIPH project

The overall strength of this study is that it enables the intervention Woredas to use their locally generated data for decision making.

Since the effectiveness of the intervention will be conducted in 12 Woredas in one zone in Amhara region, if the intervention is effective, the study findings will be further scaled-up in a wider setting.

Communication and dissemination of study findings

After the conduct of the study, study results will be disseminated through dissemination workshop by inviting the Federal Ministry of Health, North Shewa Zonal health office, Amhara Regional health bureau and other relevant stake holders.

From the final study findings, manuscript will be prepared and submitted to relevant peer reviewed scientific journals for publication.

Ethical considerations and review process

Timely ethical clearance will be sought at the Ethical Review Board of the Ethiopian Public Health Institute, and at the corresponding board at the London School of Hygiene and Tropical Medicine.

The study does not involve data-collection from community members or health records. The study participants are health-system officials, and the template of a consent form for their interviewees with a standard introduction to, and explanation of, the project is attached (Annex IV).

Voluntary participation

Participation in this study is completely voluntary and the participants are free to withdraw at any time without giving any reason and without any negative consequences.

Confidentiality

The researchers will not identify study participants by name in any reports and confidentiality as a participant in this study will remain secure. Subsequent use of data will be subject to standard data use policies that protect anonymity of study participants.

Privacy

During data collection from study participants; privacy will be secured, no one will be around. Field staff are committed to collect personal information responsibly.

Implementation of DIPH project

No	Activities											
	Year	19	19	19	19/20	20	20	20/21	21	21	21	21
	Months	May	June	Jul-Oct	Nov-Feb	Mar-Jun	Jul-Oct	Nov-Feb	Mar-Jun	July	Aug-Nov	Nov-Dec
	**Developmental phase**											
1	Ethics/IRB submission											
2	Baseline survey											
	**Implementation phase**											
3	DIPH training											
4	DIPH cycles			C1	C2	C3	C4	C5	C6			
	**Evaluation phase**											
	DIPH cycle Monitoring											
5	End-line survey											
6	Data cleaning, Analysis & reporting											
7	Dissemination											

Cost of DIPH project, budget breakdown

PERSONNEL	Year 1 (01/05/19-30/04/20)			Year 2 (01/05/20-30/04/21)			Year 3 (01/05/21-30/12/21)			Total USD for three years
	Salary per month	Annual (ETB)	Annual (USD)	Salary per month	Annual (ETB)	Annual (USD)	Salary per month	Annual (ETB)	Annual (USD)	
Salary of four DIPH support supervisors	**21,500.00**	**1,032,000.00**	**38,819.75**	**23,220.00**	**1,114,560.00**	**41,925.34**	**25,077.60**	**802,483.20**	**30,186.24**	**110,931.33**
Pension contribution	**2,365.00**	**113,520.00**	**4,270.17**	**2,554.20**	**122,601.60**	**4,611.79**	**2,758.54**	**88,273.15**	**3,320.49**	**12,202.45**
Data manger salary	**26,000.00**	**312,000.00**	**11,736.20**	**28,080.00**	**336,960.00**	**12,675.10**	**30,326.40**	**242,611.20**	**9,126.07**	**33,537.38**
Pension contribution	**2,860.00**	**34,320.00**	**1,290.98**	**3,088.80**	**37,065.60**	**1,394.26**	**3,335.90**	**26,687.23**	**1,003.87**	**3,689.11**
Regional coordintor	**33,500.00**	**402,000.00**	**15,121.65**	**36,180.00**	**434,160.00**	**16,331.38**	**39,074.40**	**312,595.20**	**11,758.59**	**43,211.62**
Pension contribution	**3,685.00**	**44,220.00**	**1,663.38**	**3,869.25**	**46,431.00**	**1,746.55**	**4,178.79**	**33,430.32**	**1,257.52**	**4,667.45**
endline - supervisor	**15,000.00**				**15,000.00**	**564.24**		**0.00**	**0.00**	**564.24**
endline - data collectors	**12,000.00**				**48,000.00**	**1,805.57**		**0.00**	**0.00**	**1,805.57**
insurance for field staffs	**40,000.00**	**40,000.00**	**1,504.64**	**42,000.00**	**42,000.00**	**1,579.87**	**44,100.00**	**44,100.00**	**1,658.87**	**4,743.38**
Total Personnel Costs	**156,910.00**	**1,978,060.00**	**74,406.79**	**138,992.25**	**2,196,778.20**	**82,634.10**	**148,851.63**	**1,550,180.30**	**58,311.65**	**215,352.53**

TRAVEL	Year 1 (01/05/19-30/04/20)				Year 2 (01/05/20-30/04/21)				Year 3 (01/05/21-30/12/21)				
	Cost	Unit	ETB	USD	Cost	Unit	ETB	USD	Cost	Unit	ETB	USD	Total USD for three years
car for Regional coordinator	267,030.00	1.00	267,030.00	10,044.61	280,381.50	1.00	280,381.50	10,546.84	294,400.58	1.00	196,267.05	7,382.79	27,974.24
**public transport for baseline field staffs**	**54,000**	1.00	54,000.00	2,031.27	56,700.00	1.00	56,700.00	2,132.83	59,535.00	1.00	39,690.00	1,492.98	5,657.08
**public transport for end line field staffs**	**4500**			0.00	4,725.00	1.00	4,725.00	177.74	0.00	1.00	0.00	0.00	177.74
**Baseline and end line field staffs training**			438,850.00	16,507.80		1.00	52,500.00	1,974.84		1.00	0.00	0.00	18,482.64
**perdiem for field staffs**	536,400.00	1.00	536,400.00	20,177.24	630,720.00	1.00	630,720.00	23,725.19	662,256.00	1.00	441,504.00	16,607.63	60,510.07
**perdiem for the drivers**	13,500.00	1.00	97,200.00	3,656.28	13,500.00	1.00	151,200.00	5,687.55	13,500.00	1.00	205,200.00	7,718.81	17,062.64
**perdiem for HR and SERO**	18,000.00	1.00	18,000.00	677.09	18,000.00	1.00	18,000.00	677.09					1,354.18
**perdiem for HR**	18,000.00	1.00	18,000.00	677.09		1.00		0.00					677.09
Total Travel Costs	861,930.00		1,296,280.00	53,771.38	1,004,026.50	8.00	1,194,226.50	44,922.07	1,029,691.58	6.00	882,661.05	33,202.21	131,895.67

OTHER DIRECT COSTS	Year 1 (01/05/19-30/04/20)				Year 2 (01/05/20-30/04/21)				Year 3 (01/05/21-30/12/21)				
	Cost	Unit	ETB	USD	Cost	Unit	ETB	USD	Cost	Unit	ETB	USD	
Office furniture	100,000.00	1.00	100,000.00	3,761.60		1.00	0.00	0.00		1.00	0.00	0.00	3,761.60
Office supplies	130,000.00	1.00	130,000.00	4,890.09	136,500.00	1.00	136,500.00	5,134.59	143,325.00	1.00	95,550.00	3,594.21	13,618.89
Dongle Apparatus	4,500.00	6.00	27,000.00	1,015.63									1,015.63
Dongle 3G internet	1,000.00	6.00	72,000.00	2,708.35	1,050.00	6.00	92,400.00	3,475.72	1,102.50	10.00	88,200.00	3,317.73	9,501.81
Mobile communications	500.00	6.00	36,000.00	1,354.18	525.00	6.00	63,000.00	2,369.81	551.25	12.00	52,920.00	1,990.64	5,714.63
Fixed phone for office	600.00		0.00	0.00	630.00	1.00	2,520.00	94.79	661.50	12.00	63,504.00	2,388.77	2,483.56
office rent	5,000.00	12.00	60,000.00	2,256.96	5,250.00	12	63,000.00	2,369.81	5,512.50	8.00	44,100.00	1,658.87	6,285.64
Total Other Direct Costs	241,600.00		425,000.00	15,986.82	143,955.00	27	357,420.00	13,444.73	151,152.75	44.00	344,274.00	12,950.23	42,381.77
**TOTAL COSTS**	1,260,440.00		3,699,340.00	144,164.98	1,286,973.75	35	3,748,424.70	141,000.90	1,329,695.96		2,777,115.35	104,464.09	389,629.97

Benefits of the Study's Results

The overall aim of the Data-Informed Platform for Health (DIPH) is to improve health programmes and services at the Woreda level. As an innovation to strengthen health systems, the DIPH promotes the sharing and use of local data for health-decision-making, particularly problem-solving. Key health-data on inputs and processes from multiple programmatic activities at the district-health administration level are brought together. This data then informs the collaborative action-plan and follow-up of different government departments and private sector agencies through a structured process that uses existing platforms at the district level. This resonates closely with the Ethiopian Health Sector Transformation Plan in 2015 [6], which identifies the information revolution as part of its transformation agenda. Promoting a culture of information-use at, or close to, the point of collection is central to the information revolution.

Importance of DIPH in Health System Strengthening

1. **Inter-sectoral convergence**, through effective coordination with key departments to address health determinants, including water, sanitation, hygiene, nutrition; education; gender and women-empowerment; and integrated Child health and Development services etc.;
2. **Multi-sectoral data-sharing;**
3. **Strengthening data-quality**, aiming to rationalize regular use of local HMIS indicators; and reliable health-data/data-triangulation mechanisms; and
4. **Accountability and planning**, through regular meetings of Performance Review Team dedicated to periodic review progress and a re-calibrated future road map, such meetings having a clear agenda and follow-up action with regular, focused reviews at different levels.

Research Collaborators

This study is a collaboration between the Ethiopian Public Health Institute (EPHI) and the London School of Hygiene and Tropical Medicine (LSHTM) under the remit of the IDEAS (Informed Decisions for Action in Maternal and Newborn Health) project. Overall, the IDEAS-EPHI team will take responsibility for the DIPH development, and for monitoring and evaluating its implementation, in active collaboration with the Health Bureau of Amhara region.

London School of Hygiene and Tropical Medicine (LSHTM)

The primary role of LSHTM is to lead the overall DIPH project coordination, provide technical support and participate in report development. This will include providing training for DIPH support supervisors, data analysis, report writing and manuscript preparation.

Ethiopian Public Health Institute

EPHI will lead the overall DIPH project implementation in terms of recruiting field staff, training, and DIPH implementation. It will also take a lead role in liaising between the DIPH project and the FMoH, Amahra regional health bureau, and similar on-going projects in the region. EPHI will be responsible for the quality of the DIPH implementation and data management. Dissemination of DIPH's findings for national and regional stakeholders

will also be one of its main roles. EPHI will also participate in providing training for support supervisors about DIPH strategy and steps. EPHI will participate in data analysis, report writing and manuscript preparation.

Amhara Health Bureau

The leading role of the Amhara Health Bureau in the DIPH project will be facilitating the project's implementation through selecting Woredas; and, from time to time, participating in field supervision during the project's implementation, enabling them to provide their views and feedback by the end of each cycle.

Assumptions, risks and mitigations

The law and order of study site, safety and security of the research staff and protection of study participants are assumed to be secured and stable. If circumstances change, research site/ location will be reconsidered. By participating in this study and answering the questions, study participants will not receive any direct benefit. However, they will help to increase the understanding of health system operations in Ethiopia. The result of this study will contribute in generating evidence and knowledge to inform policy and practice at national and global levels. This study involves provision of information through pre-developed questionnaires and the organization will keep the data in a safe place which can only be accessed by the study team. Therefore, participation in this study has minimal risk.

Facilities available for the study

The study will be conducted in EPHI where there is internet connection and a server space. The project's field office is located in Debrebirhan town where the zonal health office is located. The project will have an office based in Debrebirhan with six field staff.

Authorship right

The PI of the DIPH project and all the research team will take public responsibility for the content, concept, design, analysis, writing or revision of the study findings.

Declaration of conflict of interest

The principal investigator declared that there is no conflict of interest among co-investigators for this project.

References

1. 1. Victora CG1, Black RE, Boerma JT, Bryce J. Measuring impact in the Millennium Development Goal era and beyond: a new approach to large-scale effectiveness evaluations. Lancet. 2011 Jan 1;377(9759):85-95. doi: 10.1016/S0140-6736(10)60810-0. Epub 2010 Jul 9.
2. Heidkamp R; NEP Working Group. The National Evaluation Platform for Maternal, Newborn, and Child Health, and Nutrition: From idea to implementation. J Glob Health. 2017 Dec;7(2):020305. doi: 10.7189/jogh.07.020305.
3. Sawadogo-Lewis T. Vignola, Aung T, Heidkamp R. Developing data use capacity in the maternal, newborn, child health and nutrition sector in Malawi, Mali, Mozambique and Tanzania: an evolving strategy. J Glob Health. June 2019 • Vol. 9 No. 1 • 010310. doi: 10.7189/jogh.09.010309
3. Hanson K, Schellenberg J . Commentary- District decision making to strengthen maternal, newborn and child health servicesin low-income settings. Health Policy Plan. 2016 Sep;31 Suppl 2:ii1-ii2.
4. Wickremasinghe D, Hashmi IE, Schellenberg J, Avan BI. District decision-making for health in low-income settings: a systematic literature review. Health Policy Plan. 2016 Sep;31 Suppl 2:ii12-ii24
5. 1Bhattacharyya S, Berhanu D, Taddesse N, Srivastava A, Wickremasinghe D, Schellenberg J, Iqbal Avan B.District decision-making for health in low-income settings:a case study of the potential of publicand private sector data in India and Ethiopia. Health Policy Plan. 2016 Sep;31 Suppl 2:ii25-ii34
6. Avan BI, Berhanu D, Umar N, Wickremasinghe D, Schellenberg J. .District decision-making for health in low-income settings: a feasibility study of a data-informed platform for health in India, Nigeria and Ethiopia. Health Policy Plan. 2016 Sep;31 Suppl 2:ii3-ii11
7. Gautham M, Spicer N, Subharwal M, Gupta S, Srivastava A, Bhattacharyya S, Avan BI, Schellenberg J. District decision-making for health in low-income settings: a qualitative study in Uttar Pradesh, India, on engaging the private health sector in sharing health-related data. Health Policy Plan. 2016 Sep;31 Suppl 2:ii35-ii46
8. IDEAS-LSHTM (2018). Data Informed Platform for Health. Structured decision-making using local data. Prototype Phase, West Bengal, India. External Evaluation Report. London, UK, London School of Hygiene & Tropical Medicine.
9. Hayes RJ, Bennett S. Simple sample size calculation for cluster-randomized trials. Int J Epidemiol. 1999 Apr;28(2):319-26.

Assurance of the Principal Investigator:

I the undersigned agree to accept responsibilities for:

1. The scientific, ethical and technical conduct of the research project,
2. Requesting amendment for ANY change on the protocol that might need to happen during execution of the project, and obtain written approval for the request from EPHI-IRB,
3. Submitting progress report every year and technical report within two months after completion of the project,
4. Reporting any adverse event that might happen to the study participants, data collectors, supervisors and coordinators during investigation,
5. Submitting scientific publications that emanate from the project within two months of publication, and
6. Reporting any unprecedented protocol violation within seven days of event. if the project is approved as a result of this application.
7. Submitting your raw cleaned data to EPHI data management Center after writing the final report

Name: Bilal Avan **Signature:**

Date: 23th July 2019

Commitment for and signatures of Co-Investigators

Name **Specific Responsibility/ Signature**

From London School of Hygiene and Tropical Medicine

1. Joanna Schellenberg: Co-PI /

2. Mehret Dubale : Co investigator**/**

3. Della Berhanu: Co investigator /

4. Lars Åke Persson: Co investigator **/**

From Ethiopian Public Health Institute

1. Girum Taye: Co-PI /

2. Abebe Bekele Co investigator

Comment and concurrence of the responsible head for principal investigator

____

____

Name____________________________Signature_______________Date____________

Appendices

Annex A: Map of North Shewa within broader Amahara region.

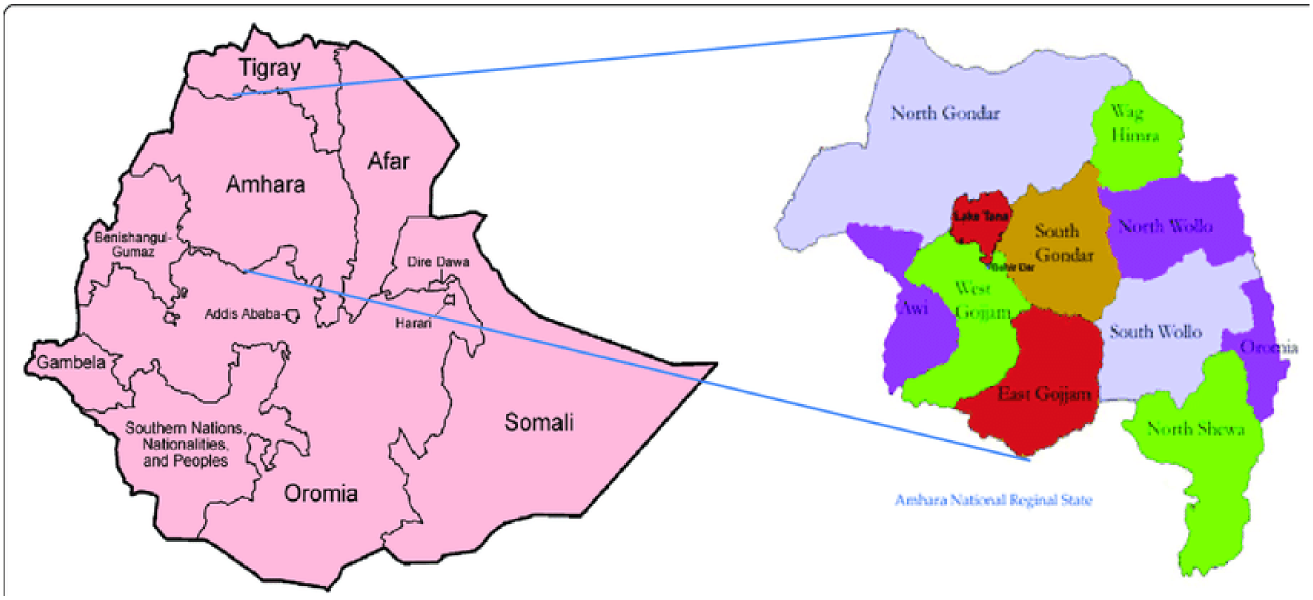

Supplementary Annex (B): List woreda by type: and population size in North Shoa zone

	Woreda Name	Population	Type
1	Minjar Shenkora	128,879	RURAL
2	Merhabete	126,501	RURAL
3	Basona Werana	120,930	RURAL
4	Menz Gera Midir	120,469	RURAL
5	Kewet	118,381	RURAL
6	Efrata Gidim	110,493	RURAL
7	Mida Woremo	93,729	RURAL
8	Moretna Jiru	92,937	RURAL
9	Menz Mama Midir	85,129	RURAL
10	Tarma Ber	84,481	RURAL
11	Angolala Tera	82,349	RURAL
12	Antsokiya Gemza	79,091	RURAL
13	Ankober	76,510	RURAL
14	Mojana Waderea	69,667	RURAL
15	Debre Berhan	65,231	URBAN
16	Gishe	61,521	RURAL
17	Saya Debirna Wayu	61,046	RURAL
18	Ensaro	58,203	RURAL
19	Hagere Mariam Kesem	55,235	RURAL
20	Asagirt	48,371	RURAL
21	Menz Keya Gebreal	46,219	RURAL
22	Berehet	34,810	RURAL
23	Shewa Robit	17,575	URBAN
24	Menz Lalo Midir	17,308	RURAL

Source: *The 2007 Population and Housing Census of Ethiopia*

Supplementary Annex (C): Health Profile of North Shewa zone on selected Maternal and Child Health indicators

Indicators	Amhara	Ethiopia
Total population[1]	21,844,000	98,665,000
Antenatal care[2]		
Mothers who had at least 3 ANC check-ups (to total ANC registered) (%)		62%
Institutional deliveries[2]		
Total institutional deliveries (as % of total reported deliveries)	27.1%	26.2%
Deliveries at public facilities (as % of total reported institutional facilities)	26.4%	24.8%
Postnatal care[2]		
Post-partum check-up within 48 hrs of delivery (as % of total reported deliveries)	12.6%	13%
Immunization coverage[2]		
% of infants fully immunized to total reported live births (BCG, 3 doses each of DPT, Polio & Measles)	46%	39%
Mortality details		
Infant mortality rate	67 per 1000	48 per 1000

Source: [1]*Central Statistical Agency population projection for Ethiopia in 2019*
[2]*The 2016 Ethiopia Demographic and Health Survey*

Supplementary Annex (D): Information about the study

DIPH: Implementation and Evaluation of Data informed Platform for health strategy in Ethiopia
INFORMED CONSENT SECTION-FRONTLINE WORKER

1. Participant Information Sheet
Good day. My name is ------------------- I am working in the research team organized by Ethiopian Public Health Institute, in collaboration with the London School of Hygiene and Tropical Medicine. I am a data collector. I would like to ask you some questions about the Woreda level decision making and data use practices. Before the questions I will provide you full information of the study so that you can decide whether or not to take part.

Project title
Study on the health of mothers, babies and children under five years of age.

Duration of the study
It will take up to two hours for each questionnaire to be fully completed.

Purpose of the Study
The overall purpose of this study is to strengthen health system at the Woreda level
With this information we will be able to give advice health ministry how to improve operations and work environment within health system at the district level.

Confidentiality
I strongly assure you your words may be quoted but the source will be shown as anonymous and they will have the chance to review any quotes used before a report is finalized.

Procedures
Specifically, I am going to ask you information about processes and work culture. You were selected to participate in this study because you are believed to have significant knowledge and experience in health system administration.

Risk and Benefits of the Study
By participating in this study and answering our questions you will not receive any direct benefit. However, you will help to increase our understanding health system operations in Ethiopia. The result of this study will contribute in generating evidence and knowledge to inform policy and practice at national and global levels. This study involves your provision of information through pre-developed questionnaires and the organization will keep your data in a safe place which can only be accessed by the study team. Therefore I want to assure you that your participation in this study has minimal risk..

Rights
Your participation in this study is voluntary and you have the right to refuse to participate or to not answer any questions that you feel uncomfortable. If you change your mind about participating during the course of the study, you have the right to withdraw at any time. The decision not to participate or to withdraw will not affect any aspect of your social life, and future medical care you should require or any other benefits to which you are entitled. If there is anything unclear or you need further information about, I am happy to provide it.

Voluntary participation

Participation in this study is completely voluntary and the participants are free to withdraw at any time without giving any reason and without there being any negative consequences.

Privacy

During data collection from study participants; no one will be around and field staff committed to collect personal information responsively.

Whom to contact

In any case if you need any information. You can contact in the following address:
The Ethiopian Public Health Institute,
Scientific and Ethical Research Office
Chairperson: Dr. Getachew Addis
Mobile Phone: 0944 123110
Address: P. O. Box 1242, Addis Ababa, Ethiopia
E-mail:get_ast@yahoo.com

Principal Investigator from EPHI side: Girum Taye
Mobile Phone: 09322223164

Address: P.O.Box 1242,Addis Ababa Ethiopia
E-mail:girumt2000@yahoo.com

2. Declaration of the Volunteer Study Participant

I understand that the purpose of the study is to collect information regarding maternal, child and newborn health. I have read the above information, or it has been read to me. I have had the opportunity to ask questions and any questions that I have asked have been answered to my satisfaction. I consent voluntarily to participate in this study and understand that I have the right to withdraw at any time without in any way affecting my social life or medical care.

Signature of Informant ________________________ Date___________________
Name of Informant___________________

- -

Signature of Witness _____________________ Name of Witness _______________________

- -

Signature of Data Collector __________________ Name of Data Collector________________

Supplementary Annex (E): DIPH Study Instruments

SECTION A: DIPH Monitoring Framework Indicators with Definitions

Module I: Utilization of data at Woreda level

Whether the DIPH study led to the utilization of the health system data or policy directive at Woreda level for decision-making?		
**Theme**	**Indicators with Definitions**	**Sources of information**
A. Selection of the primary theme for the current DIPH cycle	**1. Whether the DIPH cycle theme selection was based on HMIS data?** (1:Yes 2:No)<br><br>***Health system data:*** *statistical information collected either routinely or periodically by government institutions on public health issues. This includes information related to provision and management of health services. This data can be from the health department and/or non-health departments*<br>*In the Ethiopian context, the main data sources will include HMIS and may be non-HMIS parallel reports XXX*	Form 1B: Health system capacity assessments
	**2. Whether the DIPH cycle theme selection used any data from non-health offices?** (1:Yes 2:No)<br><br>***Non-health offices:*** *government offices, other than the health office, which directly or indirectly contributes to public health service provision*<br>*In the Ethiopian context, this includes AgricultureXXX and XXX*	Form 1B: Health system capacity assessments
	**3. Whether the DIPH cycle theme selection was based on health policy and programme directives?**(1:Yes 2:No)<br>**Health policy:** refers to decisions that are undertaken by the state/national/Woreda to achieve specific health care plans and goals. It defines a vision for the future which in turn helps to establish targets and points of reference for the short- and medium-term health programmes<br><br>***Health programme:*** *focused health interventions for a specific time period to create improvements in a very specific health domain*<br>*In the Ethiopian context: any health-related directives/guidelines/government orders in the form of an official letter or circular issued by the woreda /state government*	Form 1A: Database and document checklist
B. Data-based monitoring of the action points for the primary theme of the DIPH	**4. (Number of action points on which progress is being monitored by data) / (total number of action points for the primary theme of DIPH)**<br><br>***Action points:*** *a specific task taken to achieve a specific objective*<br>*In DIPH context: a specific action, arisen from the stakeholder discussions during Steps 3 and 4, to achieve the target of the given DIPH cycle*	Form 5: Follow-up

C. Revision of Woreda programme data elements for the primary theme of the DIPH	**5. Whether stakeholders suggested a revision/addition to health system data in the given DIPH cycle?** (1:Yes 2:No)	Form 4: Plan
	**6. (Number of data elements added in the health database as per the prepared action plan) / (total number of additional data elements requested for the primary theme of the DIPH)**<br><br>***Data elements:*** *operationally, refers to any specific information collected in the health system data forms, pertaining to all six WHO health system building blocks (demographic, human resources, finance, service delivery, health outcome, governance)*	Form 5: Follow-up
D. Improvement in the availability of health system data	**7. Whether the health system data required on the specified theme as per the given DIPH cycle was made available to the assigned person in the given DIPH cycle? (Y/N)**<br><br>***Assigned person****: as per the cycle-specific DIPH action plan; this can be the theme leader, Data Manager or any other stakeholder who is assigned with the responsibility of compiling/reporting of specified data*	Form 1B: Health system capacity assessments
	**8. Whether the health system data on the specified theme area is up-to-date as per the given DIPH cycle? (Y/N)**<br>***Up-to-date data***<br><br>1. If monthly data, then the previous complete month at the time of Step 1 of the DIPH cycle<br>2. If annual data, then the complete last year at the time of Step 1 of the DIPH cycle	Form 1B: Health system capacity assessments

Module II: Interactions among stakeholders: co-operation in decision-making, planning and implementation

Whether the DIPH study ensured involvement of stakeholders from different sectors (health, non-health and NGO/private for-profit organizations)		
**Theme**	**Indicators with Definitions**	**Sources of information**
E. Extent of stakeholder participation	**1. (Number of DIPH stakeholders present in the planning actions meeting) / (total number of DIPH stakeholders officially invited in the planning actions meeting)**<br><br>*Participants in Steps 4 and 5*<br>***DIPH stakeholders:*** *public and private sector departments, organizations and bodies relevant for the specific cycle of the DIPH*<br>***Officially invited:*** *stakeholders formally being invited to participate for the specific DIPH cycle*<br>*In the Ethiopian context, for example:*<br>1. *XXX*<br>2. *YYY*<br>3. *ZZZ*	Form A.2: Record of Proceedings – Summary Tables
	**2. (Number of representatives from the health department present in the planning actions meeting) / (total number of DIPH participants present in the planning actions meeting)**<br><br>*Participants in Steps 4 and 5*	Form A.2: Record of Proceedings – Summary Tables
	**3. (Number of representatives from non-health departments present in the planning actions meeting) / (total number of DIPH participants present in the planning actions meeting)**<br><br>*Participants in Steps 4 and 5*	Form A.2: Record of Proceedings – Summary Tables
	**4. (Number of representatives from NGOs present in the planning actions meeting) / (total number of DIPH participants present in the planning actions meeting)**<br><br>*Participants in Steps 4 and 5*	Form A.2: Record of Proceedings – Summary Tables
	**5. (Number of representatives from private for-profit organizations present in the planning actions meeting) / (total number of DIPH participants present in the planning actions meeting)**<br><br>*Participants in Steps 4 and 5*	Form A.2: Record of Proceedings – Summary Tables
F. Responsibilities assigned to stakeholders	**6. (Number of action points with responsibilities of the health department) / (total number of action points for the primary theme of the DIPH)**	Form 4: Plan
	**7. (Number of action points with responsibilities of non-health departments) / (total number of action points for the primary theme of the DIPH)**	Form 4: Plan
	**8. (Number of action points with responsibilities of NGOs) / (total**	Form 4: Plan

	**number of action points for the primary theme of the DIPH)**	
	**9. (Number of action points with responsibilities of private for-profit organizations) / (total number of action points for the primary theme of the DIPH)**	Form 4: Plan
G. Factors influencing co-operation among health, non-health and NGO/private for-profit organizations to achieve the specific action points in the given DIPH cycle	**10. List of facilitating factors**<br>**1.**<br>**2.**<br>**3**	In-Depth Interviews with Stakeholders
	**11. List of challenging factors**<br>**1.**<br>**2.**<br>**3**	In-Depth Interviews with Stakeholders
H. Action points initiated	**1. (Number of primary theme-specific action points initiated within the planned date) / (total number of primary theme-specific action points planned within the specific DIPH cycle)**	Form 5: Follow-up
I. Action points achieved	**2. (Number of primary theme-specific action points completed within the planned date) / (total number of primary theme-specific action points planned within the specific DIPH cycle)**	Form 5: Follow-up
	**3. (Number of written directives/letters issued by the Woreda/state health authority as per action plan) / (total number of written directives/letters by the Woreda/state health authority planned as per action points of the DIPH primary theme)**	Form 5: Follow-up
	**4. (Amount of finance sanctioned for the primary theme-specific action points) / (total amount of finance requested as per action points of the DIPH primary theme)**	Form 5: Follow-up
	**5. (Units of specific medicine or supplies provided for the primary theme-specific action points) / (total units of specific medicine or supplies requested as per action points of the DIPH primary theme)**	Form 5: Follow-up
	**6. (Units of specific equipment provided for the primary theme-specific action points) / (total units of specific equipment requested as per action points of the DIPH primary theme)**<br><br>***Equipment*****:** technical instruments, vehicles, etc. provided to achieve the DIPH action points	Form 5: Follow-up
	**7. (Units of specific IEC materials provided for the primary theme-specific action points) / (total units of specific IEC materials requested as per action points of the DIPH primary theme)**	Form 4: Plan<br><br>Form 5: Follow-up

	**8. (Number of human resources recruited for the primary theme-specific action points) / (total human resources recruitment needed as per action points of the DIPH primary theme)**	Form 4: Plan<br><br>Form 5: Follow-up
	**9. (Number of human resources trained for the primary theme-specific action points) / (total human resources training requested as per action points of the DIPH primary theme)**	Form 4: Plan<br>Form 5: Follow-up
J. Factors influencing the achievements as per action points of the DIPH primary theme	**10. List of facilitating factors**<br>**1.**<br>**2.**<br>**3**	In-Depth Interviews with Stakeholders
	**11. List of challenging factors**<br>**1.**<br>**2**<br>**3. .**	In-Depth Interviews with Stakeholders

SECTION B: DIPH Survey Instruments

Module I: General Identifications

1.	Facilitator ID	
1.1	Survey datedate (DD, MM, YYYY)	
1.2	Survey start time<br>(24-hour clock system, e.g., 14:30)	:
1.3	Facilitator name	
1.4	Facilitator code<br>*Enter your 2-character identifier.*	
2.	Unit ID	
**1.5**	Type of health Administration unit	1. Woreda health office<br>2. Zonal health Department<br>3. Regional health Bureau
1.6	List of the offices visited with in the admin Unit	1.<br>2.<br>3.<br>4.
1.7	Regional ID	
1.8	Zonal ID	
**1.9**	Woreda ID	
**1.10**	Respondent Designation	1= Woreda health office Head<br>2= MNCH Head/Focal Person<br>3= Data Manager<br>4= Other s, specify
**1.11**	Respondent Age (years)	
**1.12**	Gender	
**1.13**	Academic qualification	
**1.14**	Professional experience in the health department (Years)	

**1.1**	Duration of experience in the index Woreda office (months)	☐☐

Module II: Woreda profile - Resources and Potential Profiling for DIPH

1.	Staff		
2.1	Please describe the total number of people under each category below.<br>*(need further additions ..)*		
	**Title/ post**	**Number by sex**<br>(If none, enter 0; if post not applicable, write 99)	
		**Male**	**Female**
	1. Head of Woreda health office		
	2. Program officer		
	3. Disease surveillance officer		
	4. M&E/HMIS officer		
	5. Data clerk		
	96. Other (specify)____________________		

2.	Infrastructure and Equipment Inventory		
2.2	**Please verify if the following equipment or type of service is available in the facility or office.**	**A. Total quantity**<br><br>(If none, enter 0)	**B. Total quantity that are in working condition**<br>(If none, enter 0)
	1. Laptop computer		
	2. Desktop computer		
	3. Printers		
	4. Modems		
	5. Uninterruptible power supply (UPS)		
	6. Circuit breaker		
	7. Generators/grid/solar		

	8. Calculator		

checklist to assess whether or not the office has the following inventory:				
2.3	Data back-up unit			
		1. Server	1. Yes	2. No
		2. USB key	1. Yes	2. No
		3. CD (compact disc)	1. Yes	2. No
		4. External hard drive	1. Yes	2. No
		5. Zip drive	1. Yes	2. No
2.4	If yes then primary Back-up unit(s) is/are kept on site		1. Yes	2. No
2.5	1. Facility/office official mobile phone with access to telephone network		1. Yes	2. No
	2. Personal mobile phone with access to telephone network		1. Yes	2. No
2.6	1. Is there access to an Internet network?		1. Yes	2. No
	2. If *yes*, on average, how many days in a month do you have Internet access?		1. 20 days or more<br>2. 10-19 days<br>3. Less than 10 days	
	3. If yes , when was the last disruption of access (days)		☐☐	
	4. Wi-Fi		1. Yes	2. No
2.7	1. Is there a continuous electricity supply?		1. Yes	2. No
	2. If *no*, on average, how many days in a month is the electricity supply interrupted?		1. 20 days or more<br>2. 10-19 days<br>3. Less than 10 days	

3. Data sources

2.8 Data collection forms used	Source: Data collection introduced by codes:	Data collection format Codes:	Onward Reporting format Codes
	0. no form exist<br>*1. Standard HIS- FMoH*<br>*2. Programme specific-*	*0. no data collection*<br>*1. paper based*	*0. no reporting*<br>*1. paper based*

FMoH<br>*3. Regional health* Bureau<br>*4. Zonal Health Department*<br>*5. Woreda Health office*<br>*6. NGO/WHO*<br>*7. Others:*	*2. electronic*	*2. electronic*

1. Maternal health services
2. Family planning (FP) services
3. Child health services
4. Immunization services
5. General outpatient department (OPD)
6. Inpatient services
7. Tuberculosis
8. HIV/AIDS
9. Malaria
10. Other specific disease(s)
11. Nutrition services
12. Notifiable diseases/ integrated disease surveillance and response (IDSR)
13. Financial information
14. Medicines and supplies stock
15. Human resources (HR)
16. Equipment
17. Capital assets
18. Vital events
19. Other (specify)

Module III: Data Management

1.	Resources for Data Assessment	
3.1	Does the Woredahealth officehave a designated person responsible for entering data/compiling reports from health facilities?	1. Yes<br>2. No
3.3	If yes , Are designated staff trained on Data entry/compilation	1. Yes (all staff have received training in the past two years)<br>2. Mostly (all staff have received training but not in the past two years)<br>3. Partly (some staff have received training)<br>4. Not at all
3.4	Does the Woredahealth officehave written guidelines for Data entry/compilation?	1. Yes, observed<br>2. Yes, not found<br>3. No
3.5	Does the Woredahealth officehave a designated person to review the quality of compiled data prior to submission to the next level, e.g., to Zonal/regional offices, to the central health management information system (HMIS)?	1. Yes<br>2. Partly (the data are reviewed but no one is designated with the responsibility)<br>3. Not at all
3.6	If yes, Are designated staff trained on Data review and quality control	1. Yes (staff have received training in the past two years)<br>2. Mostly (all staff have received training but not in the past two years)<br>3. Partly (some staff have received training)<br>4. Not at all
3.7	Does the Woredahealth officehave written guidelines for Data review and quality control?	1. Yes, observed<br>2. Yes, not found<br>3. No

2.	Completeness of Health Facilities Reporting To Woreda For Last Three Months	
3.8	Does the Woreda keep copies of monthly data reports sent by the health facilities?<br><br>*(Check the reports from month 1 to month 3)*	1. Yes, paper-based copies only<br>2. Yes, electronic copies only<br>3. Yes, both paper-based and electronic copies (all health facilities submit both types of reports)<br>4. Yes, mixed (some health facilities submit paper-based reports; others submit electronic reports)<br>5. No

3.9	How many Primary care health facilities in the Woreda?<br>a. Health center<br>b. Health Posts<br>3. Private Clinics	

3.10	Format of monthly report to the Woredahealth officeduring last 3 months ?	**Data submission format Codes:**<br>*paper based*<br>*electronic*<br>Both	Data submission in last 3 months : 1:Yes, 2:NO<br>M-1 M-2 M-3
	a. Health centers<br>1.<br>2.<br>3.<br>b. Health posts<br>1.<br>2.<br>3.<br>c. Private clinics<br>1.<br>2.<br>3.		

3.11	If health facilities are not submitting monthly data reports, in your opinion what are the possible reasons for this?	1. Storage or archiving problems<br>2. Staffing issues<br>3. Absence of reporting forms<br>4. Transportation issues<br>5. Internet connectivity issues<br>6. Presence of other vertical reporting requirements<br>96. Other (specify)<br>____

4.	Report Timeliness	
3.12	Is there a deadline for submission of the monthly report by the health facilities?	1. Yes<br>2. No
	If *yes*, what is the Reporting deadline? : ____	
3.13	If yes, Does the Woredahealth officeoffice maintain a record of dates of receiving monthly reports?	1. Yes<br>2. No

3.14	If *yes*, how many reports were received on time (before or on the deadline)?<br>*(Check the recorded receiving dates for the three review months)*	Data submission in last 3 months on time : 1:Yes, 2:NO<br><br>M-1 M-2 M-3

Health centers
1.
2.
3.

Health posts
1.
2.
3.

Private clinics
1.
2.
3.

5.	Data Quality Assessment Mechanisms	
3.15	Does the Woredahealth officehave written guidelines on routine health data quality assessment/assurance?<br>(Observe)	1. Yes, observed<br>2. Yes, not found<br>3. No
3.16	Does the Woreda health office conduct data quality assessments at health facilities?	1. Yes<br>2. No
3.17	If *yes*, does the Woredahealth officeuse data quality assessment tools (e.g., lot quality assurance sampling [LQAS], routine data quality assessment [RDQA], in-built electronic data quality validation rules/system)?<br>[illegible]	1. Yes, observed<br>2. Yes, not found<br>3. No
3.18	Does the Woredahealth officemaintain a record of health facility data quality assessments conducted in the past 12 months? (Observe)	21. Yes, observed<br>2. Yes, not found<br>3. No
3.19	Does the Woredahealth officemaintain a record of feedback to health facilities on data quality assessment findings?<br>(Observe)	1. Yes, observed<br>2. Yes, not found<br>3. No

6.	Data Processing and Analysis	
3.20	Does the Woredahealth officeuse an electronic database/system to enter and analyze routine health data?	1. Yes<br>2. No

3.21	If *yes*, indicate the type of electronic system used for routine data entry and analysis<br><br>1. HMIS_ National proprietary software<br>2. Excel-based spreadsheet<br>3. National open-source data processing system (e.g., DHIS 2)<br>4. Other (specify)	For Data entry<br>1: yes, 2: No	For Data analysis<br>1: yes, 2: No

3.22	**Check the up-to-date reports, documents, and/or displays that contain the following information.**	
	1. Aggregated/summary HMIS report within the past three months.<br>(Observe)	1. Yes, observed<br>2. Yes, not found<br>3. No
	2. Demographic data on the catchment population of the Woreda for calculating coverages.<br>(Observe)	1. Yes, observed<br>2. Yes, not found<br>3. No
	3. Comparisons among facilities in the Woreda for key HMIS indicators. (Observe)	1. Yes, observed<br>2. Yes, not found<br>3. No
	4. Comparisons of annual Woreda/national targets.<br>(Observe)	1. Yes, observed<br>2. Yes, not found<br>3. No
	5. Comparisons of data over time (monitoring trends) for key HMIS indicators.<br>6. (Observe)	1. Yes, observed<br>2. Yes, not found<br>3. No
	7. Comparisons of service coverage along continuum of care (e.g. ANC, tetanus-toxoid [TT] immunization, facility birth etc.).<br>(Observe)	1. Yes, observed<br>2. Yes, not found<br>3. No

Module IV: Data Use Assessment Form

1.	Information Use: Guidelines Documentation	
4.1	Are there any written guidelines on Data/HMIS information display, use, and feedback?<br>(Observe)	1. Yes, copy available at the Woredahealth office<br>2. Yes, but copy not available at the Woredahealth office<br>3. No
4.2	Does the Woredahealth officehave copies of the national HMIS strategic plans, Woreda annual plans, and/or Woreda performance targets?<br>*(Observe)*	1. Yes, copy available at the Woredahealth office<br>2. Yes, but copy not available at the Woredahealth office<br>3. No

2.	Data Presentations & Visualization	
4.3	Does the Woredahealth office prepare infographics (graphs, tables, maps, etc.) showing achievements toward targets (indicators, geographic and/or temporal trends, and situation data)?*(Observe)*	1. Yes,<br>2. No
4.4	If yes, what type of information is captured in the data visuals?	
	1. Maternal health care<br>(Observe)	1. Yes<br>2. No
	2. The Expanded Program on Immunization [EPI])<br>(Observe)	1. Yes<br>2. No
	2. Neonate and child health care (other than the Expanded Program on Immunization [EPI])<br>(Observe)	1. Yes<br>2. No
	3. Top causes of morbidity and mortality<br>(Observe)	1. Yes<br>2. No
	96. Other<br>(specify)____	1. Yes<br>2. No

3.	HMIS: Analysis documentation			
**4.5**	Does the Woredahealth office have access to analyzed HMIS data (e.g., summary tables, charts, maps)?<br>*(Observe)*	1. Yes, observed paper-based<br>2. Yes, observed electronic<br>3. No		
4.6	Does the Woredahealthoffice produce any report (annual, quarterly or monthly summary/aggregate reports submitted to the higher level, etc.) based on an analysis of RHIS data?<br>*(Observe)*	1. Yes, observed<br>2. No		
4.6	If *yes*, list the reports and indicate the frequency of the reports and number of times the reports were actually issued in the past 12 months.			
	**A. Title of the report**	**B. Number of times this report is supposed to be issued per year**	**C. Number of times this report was actually issued in the past 12 months**	**D. Target audience of the report** (e.g., MOH, civil administration, parliament, community forums, general

				population)
	1.			
	2.			
	3.			

4.	Feedback to Health Facilities	
4.7	Did the Woredahealthoffice send feedback reports using Health data to health facilities in the past three months?<br>*(Observe the report and check the date)*	1. Yes, observed<br>2. No
4.9	If *yes*, indicate the types of feedback reports:	
	1. Feedback on data quality (including data accuracy, reporting timeliness, and/or report completeness)<br>*(Observe)*	1. Yes, observed<br>2. No
	2. Feedback on service performance based on reported health data (e.g., appreciation/acknowledgement of good performance; resource allocation/mobilization)<br>*(Observe)*	1. Yes, observed<br>2. No

Module V: Routine Decision-Making Forums at the Woreda Office

	A. Performance Monitoring Team Meeting	
5.1	Does the Woredahealthoffice have a Performance monitoring team (PMT) ?	1. Yes<br>2. No
5.2	If *yes*, how often are the PMT meetings supposed to take place?	1. Weekly<br>2. Monthly<br>3. Quarterly<br>4. Biannually<br>5. Annually<br>6. No schedule
5.3	When the most recent PMT was conducted? (months)	☐☐
5.4	How many times did PMT meetings take place during the past three months?	1. More than four times<br>2. Four times<br>3. Three times<br>4. Two times<br>5. One time<br>6. Not once
5.5	Were minutes of the last performance monitoring/management meetingmaintained?*(Observe)*	1. Yes<br>2. No
	If *yes*, please check the PMT meetings record for the review months and see if the following topics were discussed.	
5.6	Did they have any discussions on Health Data , such as data quality, completeness, or timeliness of reporting?<br>**(***Observe)*	1. Yes<br>2. No
5.7	If *yes*, have they made any decisions based on the discussions of health data -related issues (including no interventions required at this time)?	1. Yes<br>2. No
5.8	If *yes*, has any follow-up action taken place on the decisions made during the previous meetings on health data -related issues (e.g., referring Health data related issues/problems for solution to the higher level)?	1. Yes<br>2. No
5.9	Were discussions held to review key performance targets (tracking progress against targets) based on health data? Such as:	
	Coverage of service like ANC, delivery, EPI, or TB<br>*(Observe)*	1. Yes<br>2. No
	Hospital/health center performance indicators<br>*(Observe)*	1. Yes<br>2. No
	Disease data, e.g., top ten diseases<br>**(***Observe)*	1. Yes<br>2. No
	Identification of emerging issues/epidemics<br>*(Observe)*	1. Yes<br>2. No
	Medicine stockouts<br>(Observe)	1. Yes<br>2. No
	Human resource management<br>(Observe)	1. Yes<br>2. No
	Sex-disaggregated data<br>(Observe)	1. Yes<br>2. No

5.10	Were any decisions made based on the discussion of the Woredahealthoffice and/or health facility's performance? Such as:		
	Formulation of plans	1. Yes	2. No
	Budget preparation	1. Yes	2. No
	Budget reallocation	1. Yes	2. No
	Medicine supply and drug management	1. Yes	2. No
	Human resource management (training, reallocation, etc.)	1. Yes	2. No
	Advocacy for policy, programmatic, or strategic decisions from the higher level	1. Yes	2. No
	Health services (preventive, promotive, clinical, rehabilitative) planning	1. Yes	2. No
	Promotion of service quality/improvement	1. Yes	2. No
	Reducing the gender gap in the provision of health services	1. Yes	2. No
	Involvement of the community and local government	1. Yes	2. No
	No action required at this time	1. Yes	2. No
5.11	Were the PMT meeting minutes circulated to all members?	1. Yes<br>2. No	
5.12	Did the head of the Woreda health office attend any of the performance review/management meetings during last 6months ?	1. Yes<br>2. No	

B. Other routine health management team (HMT) meetings		
5.13	Does the Woredahealthoffice have health management team (HMT) meetings to discuss performance monitoring and management?	1. Yes<br>2. No
5.14	If *yes*, how often are the HMT meetings supposed to take place?	1. Weekly<br>2. Monthly<br>3. Quarterly<br>4. Biannually<br>5. Annually<br>6. No schedule
5.15	How many times did HMT meetings take place during the past three months?	1. More than four times<br>2. Four times<br>3. Three times<br>4. Two times<br>5. One time<br>6. Not once
5.16	Were minutes of the last health management team meetingperformancemaintained?*(Observe)*	1. Yes<br>2. No
	If *yes*, please check the HMT meetings for the review months and see if the following topics were discussed.	

5.17	Did they have any discussions on Health Data , such as data quality, completeness, or timeliness of reporting?<br>**(***Observe)*	1. Yes<br>2. No	
5.18	If *yes*, have they made any decisions based on the discussions of health data -related issues (including no interventions required at this time)?	1. Yes<br>2. No	
5.19	If *yes*, has any follow-up action taken place on the decisions made during the previous meetings on health data -related issues (e.g., referring Health data related issues/problems for solution to the higher level)?	1. Yes<br>2. No	
5.20	Were discussions held to review key performance targets (tracking progress against targets) based on health data? Such as:		
	Coverage of service like ANC, delivery, EPI, or TB<br>*(Observe)*	1. Yes<br>2. No	
	Hospital/health center performance indicators<br>*(Observe)*	1. Yes<br>2. No	
	Disease data, e.g., top ten diseases<br>**(***Observe)*	1. Yes<br>2. No	
	Identification of emerging issues/epidemics<br>*(Observe)*	1. Yes<br>2. No	
	Medicine stockouts<br>*(Observe)*	1. Yes<br>2. No	
	Human resource management<br>*(Observe)*	1. Yes<br>2. No	
	Sex-disaggregated data<br>*(Observe)*	1. Yes<br>2. No	
5.21	Were any decisions made based on the discussion of the Woredahealth office and/or health facility's performance? Such as:		
	Formulation of plans	1. Yes	2. No
	Budget preparation	1. Yes	2. No
	Budget reallocation	1. Yes	2. No
	Medicine supply and drug management	1. Yes	2. No
	Human resource management (training, reallocation, etc.)	1. Yes	2. No
	Advocacy for policy, programmatic, or strategic decisions from the higher level	1. Yes	2. No
	Health services (preventive, promotive, clinical, rehabilitative) planning	1. Yes	2. No
	Promotion of service quality/improvement	1. Yes	2. No
	Reducing the gender gap in the provision of health services	1. Yes	2. No
	Involvement of the community and local government	1. Yes	2. No

	No action required at this time	1. Yes	2. No
5.22	Were the HMT meeting minutes circulated to all members?	1. Yes<br>2. No	
5.33	Did the head of the Woreda health office attend the last health management team?	1. Yes<br>2. No	

12.	Annual Planning		
5.34	Does the Woredahealthoffice have an annual plan for the current year?	1. Yes<br>2. No	
5.33	If *yes*, does that annual plan use data from the RHIS for problem identification and/or target setting?	1. Yes<br>2. No	
5.34	If *yes*, does the annual plan contain activities and/or targets related to improving or addressing any of the following?		
	1. Coverage of service like ANC, delivery, EPI, or TB	1. Yes	2. No
	2. Hospital/health center performance	1. Yes	2. No
	3. Diseases, e.g., top ten diseases	1. Yes	2. No
	4. Emerging issues/epidemics	1. Yes	2. No
	5. Medicine stockouts	1. Yes	2. No
	6. Human resource management	1. Yes	2. No
	7. Gender disparity in health services coverage	1. Yes	2. No

**Data sharing with the non- Health Sector**		
5.35	Does the Woredahealthoffice have to submit/present health sector performance reports to the Woreda Cabinet?	1. Yes<br>2. No
5.36	If *yes*, did the Woreda submit/present health sector performance reports the Woreda Cabinet in the past one year?	1. Yes<br>2. No
5.37	Do those reports/presentations use data from the HMIS to assess the health sector’s progress?	1. Yes<br>2. No

Module VI: Decision making Culture

We would like to know your opinion (regarding certain aspects of the Health system decision making i.e how strongly you agree or disagree with the given option. The scale assesses the intensity of your belief and ranges from "strongly disagree"(score of 1)to "strongly agree"(score of 5).There is no right or wrong answer, only an expression of your opinion based on a scale

**6.1**	**In the woreda health office , decisions are based on:**	**Strongly disagree**	**Disagree**	**Neither disagree nor agree**	**Agree**	**Strongly agree**
	Personal preference of decision makers	1	2	3	4	5
	Superiors' directives	1	2	3	4	5
	Evidence/facts/data	1	2	3	4	5
	History (e.g., what was done last year)	1	2	3	4	5
	Funding directives from higher levels	1	2	3	4	5
	Political considerations	1	2	3	4	5
	Official health sector strategic objectives	1	2	3	4	5
	Locally identified health needs of the population	1	2	3	4	5
	The relative cost of interventions	1	2	3	4	5
	Participatory decision making, by obtaining input from relevant staff	1	2	3	4	5

**6.2**	**In the health department, superiors (managers or higher-level supervisors):**	**Strongly disagree**	**Disagree**	**Neither disagree nor agree**	**Agree**	**Strongly agree**
	Seek input from the relevant woreda health office staff	1	2	3	4	5
	Emphasize that data quality procedures be followed in the compilation and submission of periodic reports (e.g., monthly reports)	1	2	3	4	5
	Promote multidirectional feedback mechanisms to share/present information within the team, and to lower and upper levels of the health system	1	2	3	4	5
	Use RHIS data for service performance monitoring and target setting	1	2	3	4	5
	Emphasize the need to use RHIS data to identify potential gender-related disparities in service delivery or use	1	2	3	4	5
	Conduct routine data quality checks at points where data are captured, processed, or aggregated	1	2	3	4	5
	Ensure that regular meetings are held where data and information are discussed, performance reports are presented and reviewed, decisions are made, follow-up actions are identified, and their implementation is monitored	1	2	3	4	5
	Provide regular feedback on reported data quality (e.g., accuracy of data compilation/reporting) to the staff responsible for compiling and reporting the data	1	2	3	4	5
	Recognize or reward staff for good work performance	1	2	3	4	5

6.3	In the health department staff:	Strongly disagree	Disagree	Neither disagree nor agree	Agree	Strongly agree
	Complete RHIS tasks (reporting, processing/aggregation, and/or analysis) in a timely manner (i.e., meet appropriate deadlines)	1	2	3	4	5
	Display commitment to the RHIS mission (i.e., to generate and use good-quality—accurate, complete, and timely—data for evidence-based decision making)	1	2	3	4	5
	Pursue national targets and set feasible local targets for essential service performance	1	2	3	4	5
	Feel "personal responsibility" for failing to reach performance targets	1	2	3	4	5
	Use RHIS data for day-to-day management of the facility and Woreda (e.g., service delivery, financial, commodities, and human resource management)	1	2	3	4	5
	Use RHIS data to solve common problems in service delivery	1	2	3	4	5
	Use sex-disaggregated or gender-sensitive RHIS data to identify and/or solve gender-related problems in service delivery	1	2	3	4	5
	Prepare data visuals (graphs, tables, maps, etc.) showing progress toward targets (indicators, geographic and/or temporal trends, or situation data)	1	2	3	4	5
	Can evaluate whether an intervention achieved the target(s) or goal(s)	1	2	3	4	5
	Are able to make decisions appropriate to their job descriptions in response to the findings of data analysis (e.g., changes in service delivery or management practices)	1	2	3	4	5
	Are held accountable for poor performance (e.g., failure to meet reporting deadlines)	1	2	3	4	5
	Admit mistakes if/when they occur and take corrective action	1	2	3	4	5

6.4	**Personal feelings:**	**Strongly disagree**	**Disagree**	**Neither disagree nor agree**	**Agree**	**Strongly agree**
	I feel discouraged when the data that I collect/record are not used for taking action (either for monitoring or decision making)	1	2	3	4	5
	I find collecting/recording data to be tedious (i.e., repetitive or duplicative)	1	2	3	4	5
	I find that the data that I collect burdens my workload, making it difficult for me to complete my other duties	1	2	3	4	5
	Collecting data is meaningful/useful for me	1	2	3	4	5
	I feel that the data I collect are important for monitoring the performance of the health services provided at my facility/unit	1	2	3	4	5
	My work of collecting data is appreciated and valued by supervisors	1	2	3	4	5
	I feel that data collection/recording is not the responsibility of healthcare providers	1	2	3	4	5

References

1Victora CG1, Black RE, Boerma JT, Bryce J. Measuring impact in the Millennium Development Goal era and beyond: a new approach to large-scale effectiveness evaluations. Lancet. 2011 Jan 1;377(9759):85-95. doi: 10.1016/S0140-6736(10)60810-0. Epub 2010 Jul 9.

2Heidkamp R; NEP Working Group. The National Evaluation Platform for Maternal, Newborn, and Child Health, and Nutrition: From idea to implementation. J Glob Health. 2017 Dec;7(2):020305. doi: 10.7189/jogh.07.020305.

3Sawadogo-Lewis T. Vignola, Aung T, Heidkamp R. Developing data use capacity in the maternal, newborn, child health and nutrition sector in Malawi, Mali, Mozambique and Tanzania: an evolving strategy. J Glob Health. June 2019 • Vol. 9 No. 1 • 010310. doi: 10.7189/jogh.09.010309

4Hanson K, Schellenberg J . Commentary- District decision making to strengthen maternal, newborn and child health servicesin low-income settings. Health Policy Plan. 2016 Sep;31 Suppl 2:ii1-ii2.

5Wickremasinghe D, Hashmi IE, Schellenberg J, Avan BI. District decision-making for health in low-income settings: a systematic literature review. Health Policy Plan. 2016 Sep;31 Suppl 2:ii12-ii24

6Bhattacharyya S, Berhanu D, Taddesse N, Srivastava A, Wickremasinghe D, Schellenberg J, Iqbal Avan B.District decision-making for health in low-income settings:a case study of the potential of publicand private sector data in India and Ethiopia. Health Policy Plan. 2016 Sep;31 Suppl 2:ii25-ii34

7Avan BI, Berhanu D, Umar N, Wickremasinghe D, Schellenberg J. .District decision-making for health in low-income settings: a feasibility study of a data-informed platform for health in India, Nigeria and Ethiopia. Health Policy Plan. 2016 Sep;31 Suppl 2:ii3-ii11

8Gautham M, Spicer N, Subharwal M, Gupta S, Srivastava A, Bhattacharyya S, Avan BI, Schellenberg J. District decision-making for health in low-income settings: a qualitative study in Uttar Pradesh, India, on engaging the private health sector in sharing health-related data. Health Policy Plan. 2016 Sep;31 Suppl 2:ii35-ii46

9 IDEAS-LSHTM (2018). Data Informed Platform for Health. Structured decision-making using local data. Prototype Phase, West Bengal, India. External Evaluation Report. London, UK, London School of Hygiene & Tropical Medicine.

10 Hayes RJ, Bennett S. Simple sample size calculation for cluster-randomized trials. Int J Epidemiol. 1999 Apr;28(2):319-26.
